# Advancements in photosynthetic efficiency: Pathways, regulation, and biotechnological applications for enhancing crop productivity

**DOI:** 10.1080/15592324.2025.2596483

**Published:** 2025-12-04

**Authors:** Tanveer Alam Khan, Sunil Mundra, Mayank Anand Gururani

**Affiliations:** a Department of Biology, College of Science, United Arab Emirates University, Al Ain, United Arab Emirates

**Keywords:** Photosynthetic efficiency, RuBisCO, ATP synthase, CRISPR/Cas9, C₄ engineering, redox regulation, crop productivity

## Abstract

Photosynthesis defines the upper limit of crop productivity, yet intrinsic inefficiencies in light capture, carbon fixation, and energy conversion constrain yield potential under variable environmental conditions. This review provides a mechanistic synthesis of recent advances in enhancing photosynthetic efficiency through molecular, biochemical, and biophysical strategies. We highlight key regulatory processes governing RuBisCO activity, ATP synthase function, photosystems, and light-harvesting complexes, together with emerging insights into redox modulation, photorespiration, and post-translational control. Innovations in genome editing, particularly CRISPR/Cas9, synthetic biology, and systems modeling, are accelerating the rational redesign of photosynthetic pathways to improve carbon assimilation and stress resilience. Engineering C₄ and CAM traits into C₃ crops, optimizing canopy light utilization, and modifying photoprotective and photorespiratory pathways demonstrate substantial potential to overcome long-standing biochemical and anatomical constraints. Integration of high-throughput phenotyping, multi-omics analysis, and computational modeling is now enabling predictive frameworks for photosynthetic improvement under fluctuating light, temperature, and water regimes. Coupling these molecular innovations with stress-tolerance traits such as enhanced antioxidant capacity and water-use efficiency offers a viable path toward climate-resilient, high-yield crops. Collectively, these advances illustrate how precise manipulation of photosynthetic processes can drive sustainable gains in agricultural productivity to meet future global food demand.

## 1 Introduction

Photosynthetic performance in crops is governed by a complex interplay of intrinsic and extrinsic factors that collectively determine carbon assimilation efficiency and yield potential. Among the intrinsic determinants, stomatal and mesophyll conductance regulate CO₂ diffusion to the chloroplasts, while biochemical processes involving RuBisCO activity and the Calvin–Benson cycle drive carbon fixation. In parallel, the efficiency of thylakoid electron transport and mechanisms such as non-photochemical quenching are essential for maintaining energy balance, protecting the photosystems, and sustaining optimal carbon metabolism under variable light conditions.^1^^,^^2^ Genetic manipulation and the incorporation of photosynthetic mechanisms from algae or cyanobacteria into plant chloroplasts represent promising strategies for enhancing photosynthetic performance.^3^ Optimizing photosynthesis under fluctuating light conditions is particularly critical, as modern crop canopies experience dynamic light environments that can reduce CO₂ assimilation by 10–40% due to the slow acclimation of photosynthetic processes.^4^ Transgenic interventions that accelerate these acclimation responses have demonstrated potential to improve productivity.^4^ In C₄ crops, increasing RuBisCO content and electron transport capacity can further enhance photosynthesis under both optimal and stress conditions by alleviating bottlenecks in the NADP-malic enzyme pathway.^5^ Moreover, the conversion of C₃ crops into C₄ plants offers a viable strategy for sustaining productivity in hot and arid environments.^3^ Environmental factors, including light intensity, temperature, and atmospheric CO₂, play decisive roles in regulating photosynthetic efficiency. A detailed understanding of their mechanistic interactions is essential for developing adaptive strategies.^2^ Recent advances in gene editing and environmental modeling have enabled the identification and manipulation of key control points in photosynthesis, paving the way for significant improvements in crop productivity.^6^ In parallel, enhancing the conversion efficiency of light energy into biomass through targeted modifications of photoprotective and photorespiratory pathways presents additional opportunities to improve photosynthetic efficiency.^7^ Collectively, integrating genetic, biochemical, and environmental approaches offers a robust framework for optimizing photosynthesis to meet the rising global demand for food.^8^

Global food security is increasingly threatened by population growth, climate change, and unsustainable agricultural practices. With the world population projected to approach 10 billion by 2050, achieving sustainable growth in food production is imperative.^9^ Climate change compounds these pressures by intensifying droughts, accelerating soil degradation, and increasing the frequency of extreme weather events, all of which constrain agricultural productivity.^10^ Additionally, geopolitical disruptions, such as the war in Ukraine, have further destabilized global food supply chains, causing shortages and escalating prices.^10^ Addressing these multifaceted challenges demands innovative, sustainable, and scalable strategies to enhance agricultural resilience. Biotechnological innovations, including genetic modification, molecular breeding, and precision management practices, can substantially improve yields of staple crops such as wheat, rice, and maize, the keystone of global nutrition.^11^ Integrating ecohydrological principles and optimizing green water management within the root zone further contributes to climate-resilient farming systems.^12^ At the policy level, systemic reforms are required to eliminate inefficiencies and incentivize sustainability across agricultural value chains.^13^ A holistic approach, combining technological innovation, evidence-based policy, and international cooperation, is therefore vital for achieving long-term food security.^14^ Such an approach must also promote responsible innovation, equitable resource distribution, and environmental stewardship,^15^ ensuring that productivity gains do not come at the expense of ecological integrity.^16^^,^^17^

The primary objective of this review is to provide a mechanistic synthesis of recent advances in photosynthesis research, emphasizing its central importance in addressing global food-security challenges. We consolidate current understanding of photosynthetic pathways, regulatory networks, and the physiological constraints that limit efficiency, while critically evaluating emerging genetic and biotechnological strategies aimed at improving crop productivity. Particular attention is given to recent progress in molecular and genetic regulation, including CRISPR/Cas9-mediated genome editing, high-throughput phenotyping, and computational modeling, which enables precise manipulation of photosynthetic traits. We also examine how photosynthetic enhancement can be integrated with abiotic stress tolerance mechanisms to sustain yields under drought, heat, and other environmental extremes. Finally, we identify key research priorities and highlight the necessity of interdisciplinary collaboration to fully realize the transformative potential of photosynthetic optimization for sustainable agriculture and climate resilience.

## 2 Photosynthetic efficiency: fundamental concepts

### 2.1 Key components: light capture, energy conversion, and carbon assimilation,

**Figure 1.**
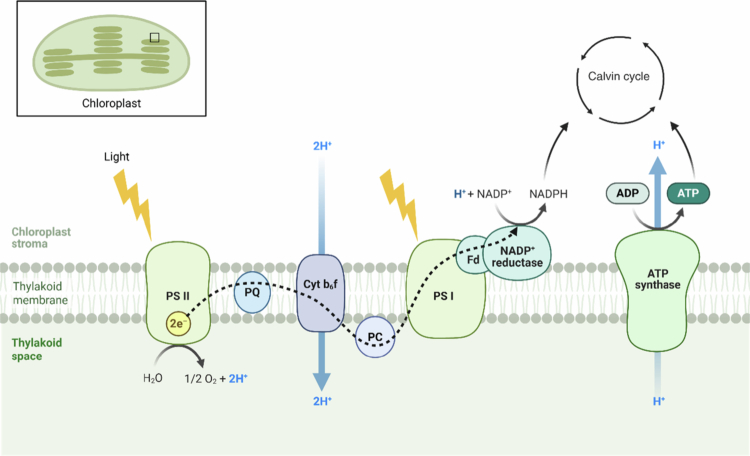
Light-dependent reactions of photosynthesis illustrating light capture and energy conversion in the thylakoid membrane. Light energy absorbed by PSII and PSI initiates electron transfer through plastoquinone (PQ), cytochrome b_₆_f (Cyt b_₆_f), and plastocyanin (PC), resulting in the production of NADPH and ATP. Water oxidation at PSII releases molecular oxygen (O₂) and protons into the thylakoid lumen, establishing a proton gradient that drives ATP synthase. The ATP and NADPH generated in these reactions provide the energy and reducing power required for carbon assimilation in the Calvin cycle.

Photosynthesis is a complex process involving three key components: light capture, energy conversion, and carbon assimilation (Figure 1). The figure illustrates the light-dependent reactions occurring in the thylakoid membrane, where light capture and energy conversion take place. Light capture is mediated by photosynthetic pigments such as chlorophylls, carotenoids, and phycobilins, which absorb light across a range of wavelengths, enabling plants, algae, and cyanobacteria to adapt to varying light environments.^18^ The absorbed light energy is transferred through antenna complexes to the reaction centers of PS II and PSI, where it is converted into chemical energy with high efficiency.^19^ As shown in Figure 1, PSII uses absorbed light to split water molecules, releasing oxygen, electrons, and protons. The electrons travel through an electron transport chain involving plastoquinone (PQ), cytochrome b₆f (Cyt b₆f), and plastocyanin (PC), generating a proton gradient across the thylakoid membrane. PSI further energizes the electrons using light energy and transfers them to ferredoxin (Fd) and NADP⁺ reductase, leading to the production of NADPH. The proton gradient drives ATP synthesis via ATP synthase, providing ATP and NADPH, the essential energy carriers for carbon assimilation in the Calvin cycle.^20^ Carbon assimilation subsequently occurs in the stroma, where CO₂ is fixed into organic molecules through the Calvin–Benson cycle. This process is further enhanced by mechanisms such as C₂ photosynthesis, which improves CO₂ re-assimilation during photorespiration.^21^ Together, these tightly integrated processes of light capture, energy conversion, and carbon assimilation sustain life on Earth and inspire innovations in artificial photosynthesis for renewable energy generation and carbon capture.^22-25^

### 2.2 C_4_ Photosynthesis—mechanism and engineering

**Figure 2.**
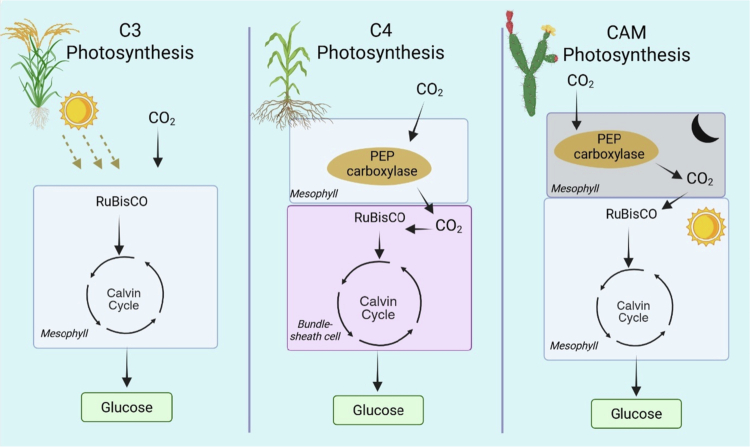
Overview of photosynthetic pathways (C_*3*_*,* C_4_, and CAM): In C₃ plants, CO₂ is directly fixed by RuBisCO in the mesophyll cells, initiating the Calvin cycle to produce glucose. C₄ plants involve spatial separation of carbon fixation and the Calvin cycle: CO₂ is initially fixed by phosphoenolpyruvate (PEP) carboxylase in the mesophyll and then delivered as a C₄ compound to bundle sheath cells, where RuBisCO operates with reduced photorespiration. CAM plant features temporal separation: CO₂ is fixed at night by PEP carboxylase and stored as organic acids, which are decarboxylated during the day to release CO₂ for the Calvin cycle in the same mesophyll cells. Each pathway represents an adaptive strategy to optimize carbon fixation under varying environmental conditions.

Plants employ three canonical photosynthetic strategies, C_3_, C_4_, and CAM, distinguished by enzyme usage and by the spatial or temporal organization of carbon fixation. In C_3_ photosynthesis, the most common form, RuBisCO fixes CO₂ directly via the Calvin cycle within mesophyll cells (Figure 2). This pathway performs well under cool, moist conditions with moderate light but becomes inefficient in hot, dry environments because photorespiration increases when RuBisCO fixes O₂, wasting energy and reducing carbon gain.^26^^,^^27^ By contrast, C_4_ photosynthesis, evolved multiple times from C_3_ ancestors, spatially separates initial CO₂ capture from the Calvin cycle across cell types: phosphoenolpyruvate carboxylase (PEPC) in mesophyll cells fixes CO₂ into a four-carbon acid that is transported to bundle-sheath cells, where CO₂ is released and refixed by RuBisCO (Figure 2). This spatial partitioning suppresses photorespiration and enhances efficiency under high light, high temperature, and low CO₂.^28^^,^^29^ CAM photosynthesis instead uses temporal separation: stomata open at night to fix CO₂ with PEPC into organic acids, which are stored and decarboxylated during the day to supply CO₂ to RuBisCO while stomata remain closed to conserve water (Figure 2). This strategy underpins high water-use efficiency and success in arid habitats; moreover, many CAM species exhibit a C_3_–CAM continuum that reflects evolutionary flexibility.^30-34^ While both C_4_ and CAM deploy PEPC, C_4_ relies on spatial partitioning, whereas CAM relies on temporal partitioning.

**Figure 3.**
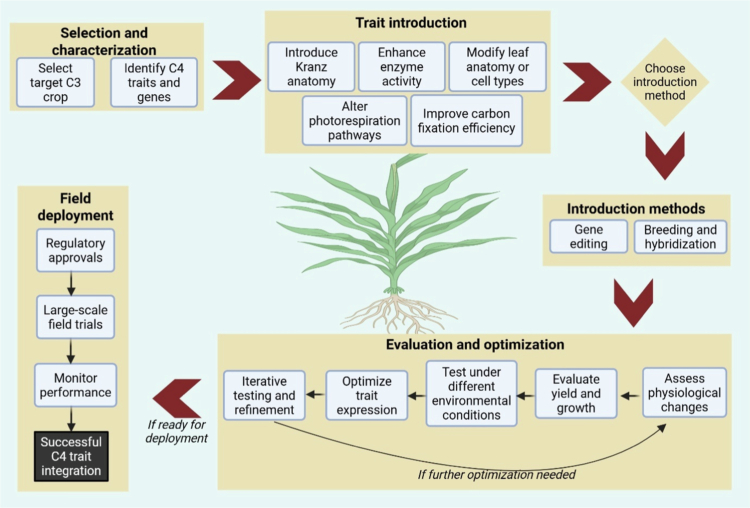
Engineering C_4_ traits into C_3_ crops: The process begins with the selection of a target C₃ crop and the identification of desirable C₄ traits and genes. Trait introduction involves strategies such as incorporating Kranz anatomy, enhancing enzyme activity, modifying leaf or cell anatomy, altering photorespiration, and improving carbon fixation efficiency. These traits are introduced using gene editing or breeding approaches, followed by evaluation of physiological, agronomic, and environmental performance. Iterative optimization ensures trait stability. Once validated, the crop proceeds through regulatory approvals, field trials, and performance monitoring for deployment.

In addition to this, human population growth continues to outpace yield gains; multiple, complementary strategies are required to meet future food demand. Engineering C_4_ traits into C_3_ crops is a leading avenue to raise productivity and resource-use efficiency at scale (Figure 3).

The C_4_ carbon-concentrating mechanism (CCM) elevates CO₂ around RuBisCO, curbing photorespiration and improving photosynthetic rates, particularly under the high light, heat, and episodic water limitation typical of many production regions.^35^^,^^36^ Modeling and empirical studies suggest that installing C_4_ features in C_3_ staples such as rice could substantially increase carbon assimilation, approaching a doubling in some scenarios, thereby accelerating growth and enhancing drought performance.^35^^,^^37^ Beyond higher carboxylation efficiency, C_4_ plants often achieve superior radiation-use, water-use, and nitrogen-use efficiencies relative to C_3_ plants, pointing to a route past current biomass ceilings.^38^ Concentrating CO₂ at the site of RuBisCO reduces photorespiratory losses, a dominant limitation in many C_3_ crops, and can sustain productivity with lower water and nitrogen inputs, advantages expected to become increasingly valuable under warming climates.^37^^,^^39-41^ Realizing these gains, however, requires coordinated changes in leaf anatomy, biochemistry, and gene regulation to recreate the hallmark spatial separation of C_4_ photosynthesis. This includes establishing Kranz-like anatomy and partitioning PEPC, decarboxylases, transporters, and Calvin cycle activities between mesophyll and bundle-sheath cells under appropriate regulatory control.^39^^,^^42^ Recent advances in genetic and evolutionary engineering, such as identifying C_4_-enriched genes and regulatory modules and designing synthetic pathways including the *β*-hydroxy aspartate cycle, provide a growing toolkit to address these requirements.^38^^,^^41^ Equally important is leveraging natural variation and evolutionary intermediates along the C_3_→C_4_ trajectory to prioritize tractable targets and stage the transition in manageable steps.^40^^,^^43^ When integrated with modern breeding and systems biology, strategic incorporation of C_4_ traits into C_3_ crops offers a credible path to higher, more resource-efficient yields and greater resilience, supporting global food security amid climate change and population growth.^42^^,^^44^

## 3 Advances in understanding photosynthetic mechanisms

### 3.1 Molecular and genetic regulation of photosynthesis

Recent research on the molecular and genetic regulation of photosynthesis has explored diverse aspects, including genetic engineering, transcription factors, regulatory networks, and post-translational modifications. These studies aim to enhance photosynthetic efficiency, a critical factor for increasing crop yields and addressing global food security challenges. Investigations span a range of organisms, from model plants such as *Arabidopsis thaliana* to trees like *Populus tomentosa* and algae including *Chlamydomonas reinhardtii*. Notably, genetic manipulation of photosynthetic pathways, particularly the Calvin cycle and electron transport chain, has demonstrated the potential to increase crop yields by over 40% under both controlled and field conditions.^18^

The exploitation of natural genetic variation in transcription factors (TFs) and photosynthesis-related genes has been shown to regulate photosynthetic traits, providing a pathway to improve photosynthetic efficiency through marker-assisted selection.^45^ Transcription factors play a crucial role in regulating photosynthesis by controlling the expression of genes involved in light reactions and other photosynthetic processes. For instance, a SNP in the zf-Dof 5.6 gene affects photosynthesis by modulating a network of TFs that regulate numerous photosynthesis-related genes.^45^ In *Arabidopsis thaliana*, a gene regulatory network involving TFs has been reconstructed, revealing a "small world" property that suggests substantial coordination between different photosynthetic components.^46^ Post-translational modifications (PTMs) such as phosphorylation, acetylation, and redox regulation play significant roles in modulating photosynthetic processes. These modifications impact the distribution of light energy between photosystems and the efficiency of carbon assimilation.^47^ Recent advances have identified various PTMs in chloroplast proteins, which regulate DNA replication, transcription, and metabolic activities, highlighting the complexity of photosynthetic regulation.^47^

Photosynthetic efficiency is influenced by environmental factors such as light quality, temperature, and CO_2_ concentration. Organisms like *Chlamydomonas reinhardtii* exhibit dynamic regulation of photosynthesis in response to these factors, involving state transitions and cyclic electron flow.^48^ Site-specific regulation of photosynthesis, where different modes of photosynthesis may occur in different plant organs or habitats, offers potential for enhancing yields in C_3_ crops like wheat and rice.^49^ Moreover, genome-wide association studies have identified genomic regions and genes associated with photosynthesis energy partitioning, particularly in rice. These studies highlight the importance of specific chromosomes in regulating photochemistry processes.^50^ The coordinated regulation of photosynthesis and wood formation in *Populus tomentosa* has been explored, revealing pleiotropic genes that balance these processes, which is crucial for tree breeding and ecosystem services.^51^

The complexity of regulatory networks and the influence of environmental factors necessitate further research to fully harness the potential of photosynthetic improvements for agricultural applications. Additionally, the integration of genetic and environmental data could lead to more robust strategies for enhancing photosynthetic efficiency across diverse plant species.

### 3.2 Chlorophyll and light capture

The relationship between chlorophyll content and light capture in photosynthetic organisms is influenced by several key factors, including the type and abundance of photosynthetic pigments, the structural organization of light-harvesting complexes, and the environmental conditions to which the organisms are exposed. Chlorophyll, along with carotenoids and phycobilins, plays a crucial role in light absorption, with each pigment type adapting to different light environments to optimize photosynthesis.^18^ Interestingly, a study on a chlorophyll-deficient rice mutant demonstrated that reduced chlorophyll content does not necessarily limit light capture or photosynthetic efficiency, as the mutant compensated for lower chlorophyll levels with a higher quantum yield of PSII and efficient electron transport.^52^ This suggests that the efficiency of light capture is not solely dependent on chlorophyll quantity but also on the organism's ability to optimize the use of absorbed light. Additionally, the modulation of chlorophyll fluorescence in response to light intensity and spectrum variations highlights the dynamic nature of photosynthetic efficiency, which can be affected by environmental light conditions.^53^ The integration of chlorophyll fluorescence data into metabolic models has further enhanced the understanding of light-driven metabolism, revealing strategies such as non-photochemical quenching and energy dissipation that organisms use to acclimate to varying light conditions.^54^ In dense plant canopies, engineering the size and composition of light-harvesting antennae, including chlorophyll content, has been proposed to improve light distribution and photosynthetic efficiency, particularly in crowded environments where light is a limiting factor.^55^ Moreover, photosynthetic organisms exhibit remarkable adaptability, with different species employing unique strategies to optimize light capture according to their ecological niches.^56^ The regulation of light-harvesting systems, including the dynamic adjustment of antenna sizes and the use of far-red photons, further illustrates the complexity of light capture mechanisms and their impact on photosynthetic efficiency and biomass yield.^57-59^ Overall, the relationship between chlorophyll content and light capture is a multifaceted interaction influenced by pigment composition, structural adaptations, and environmental acclimation strategies.

### 3.3 Photosystems and electron transport

Photosystems and electron transport chains are integral to regulating photosynthetic efficiency in plants through a complex interplay of linear and cyclic electron transport pathways. PSI and PSII work in tandem to drive the photosynthetic electron transport chain, with PSI being particularly vulnerable to photoinhibition under imbalanced conditions, such as high light or low temperatures, which can severely impact carbon fixation and plant growth.^60^ Regulation of electron transport is critical for maintaining photosynthetic efficiency, as it ensures a proper balance between ATP and NADPH production via both linear and cyclic pathways. Cyclic electron transport around PSI, mediated by the proton gradient regulation 5 (PGR5) complex, plays a key role in generating ATP without producing NADPH, thereby maintaining the ATP/NADPH ratio and protecting the photosystems from over-reduction.^61^^,^^62^ This cyclic pathway is particularly important under dynamic environmental conditions, where it helps modulate electron flow and protect PSI from damage.^63^ Additionally, state transitions, which involve the redistribution of excitation energy between PSI and PSII, play a role in optimizing photosynthetic efficiency by ensuring balanced excitation of the photosystems, although their impact on efficiency in higher plants is still being elucidated.^64^ The regulation of the electron transport chain is further influenced by the interaction of ferredoxin: NADP(H) oxidoreductase (FNR) with specific proteins, which affects the activity of alternative electron transport pathways during transitions from dark to light.^65^ Moreover, the proton motive force across the thylakoid membrane, regulated by cyclic and non-cyclic electron transport, is crucial for ATP synthesis and non-photochemical quenching, which are vital under conditions where metabolism is limiting.^66^ These regulatory mechanisms collectively ensure that photosynthesis is efficient and adaptable to varying environmental conditions, thereby supporting plant growth and productivity.

### 3.4 RuBisCO and strategies to minimize photorespiration

**Figure 4.**
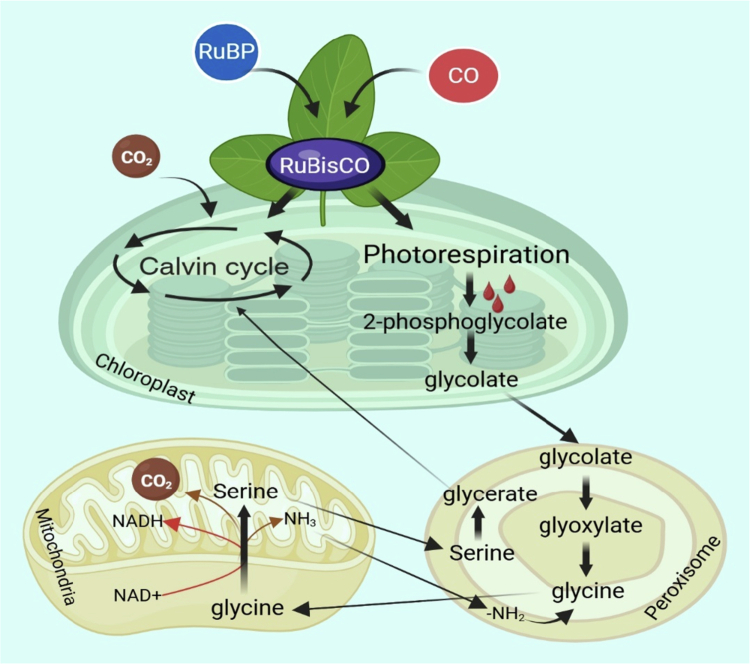
Role of RuBisCO and photorespiration in C₃ plants: RuBisCO catalyzes both carboxylation and oxygenation of RuBP. The oxygenation pathway produces 2-phosphoglycolate, which enters the photorespiratory cycle involving chloroplasts, peroxisomes, and mitochondria. Through a series of metabolic conversions, including glycolate to glyoxylate, glycine to serine, this pathway results in CO₂ and NH₃ release, representing a loss of fixed carbon and nitrogen, and reducing photosynthetic efficiency.

RuBisCO, or ribulose−1,5-bisphosphate carboxylase/oxygenase, plays a pivotal role in regulating photorespiration in plants through its dual enzymatic activity, which involves both carboxylation and oxygenation reactions. The oxygenation reaction of RuBisCO leads to the production of 2-phosphoglycolate, a compound that inhibits photosynthetic carbon fixation and must be rapidly metabolized through the photorespiratory pathway to prevent metabolic collapse.^67^^,^^68^ This pathway, which spans chloroplasts, peroxisomes, and mitochondria, recycles 2-phosphoglycolate into 3-phosphoglycerate, allowing the Calvin cycle to continue in the presence of oxygen (Figure 4),^67^^,^^69^. The regulation of photorespiration is complex and involves several molecular mechanisms. For instance, glycine decarboxylase (GDC) is a key enzyme in the photorespiratory cycle, and its activity is modulated by redox regulation through mitochondrial thioredoxin o1 (TRXo1), which adjusts the photorespiratory flux in response to environmental changes.^70^ Additionally, the interaction between photorespiration and other metabolic pathways, such as nitrogen assimilation and redox metabolism, highlights its integrative role in plant metabolism.^71^^,^^72^ The kinetic properties of RuBisCO, including its specificity for CO_2_ over O_2_, are crucial for the evolution of carbon concentrating mechanisms like the C_4_ pathway, which suppresses photorespiration by increasing CO_2_ concentrations around RuBisCO.^73^ Moreover, the regulation of RuBisCO itself involves the binding of sugar phosphate derivatives, which inhibit its activity and are removed by RuBisCO activase to restore catalytic competency.^74^ Understanding these molecular mechanisms is essential for optimizing photosynthesis and improving plant growth under varying environmental conditions.^68^^,^^75^ Overall, RuBisCO's regulation of photorespiration is a multifaceted process that balances carbon fixation and energy efficiency, crucial for plant adaptation and productivity.^76^

### 3.5 ATP synthase and energy conversion

ATP synthase plays a pivotal role in photosynthetic efficiency by converting the proton motive force (pmf) generated during photosynthesis into ATP, which is essential for various metabolic processes in plants. The efficiency of this conversion is influenced by the structure of the ATP synthase complex, particularly the c-ring stoichiometry, which determines the H^+^/ATP ratio.^77^ In photosynthetic organisms, a high H^+^/ATP ratio is observed, which, while resulting in a low ATP/NADPH ratio, helps maintain pmf within a range that supports ATP synthesis without causing photodamage due to excess electric gradient (Δψ) and pH concentration gradient (ΔpH).^77^ Engineering efforts, such as altering the c-ring stoichiometry in tobacco chloroplasts, have shown that increasing the number of c-subunits can enhance the membrane potential contribution to pmf, thereby maintaining efficient ATP synthesis even with reduced ATP synthase abundance.^78^ Additionally, genetic modifications, such as the overexpression of the AtpD subunit in rice, have been shown to increase the abundance and activity of ATP synthase, thereby enhancing photosynthetic electron transport under high light and CO_2_ conditions.^79^ Furthermore, specific mutations in ATP synthase subunits, like the C252Y mutation in *Synechococcus elongatus*, have been linked to improved stress tolerance and increased ATP synthase activity, highlighting the enzyme's role in stress acclimation and energy conversion efficiency.^80^ These findings underscore the potential of ATP synthase as a target for improving photosynthetic efficiency through synthetic biology and genetic engineering, offering promising avenues for enhancing crop yields and stress resilience in the face of changing environmental conditions.^81-83^

### 3.6 Nuclear and chloroplast genes

The regulation of photosynthetic efficiency in plants is a complex process involving the coordinated expression of nuclear and chloroplast genes. Chloroplasts, which evolved from ancient cyanobacterial endosymbionts, retain a reduced genome that works in concert with the nuclear genome to facilitate photosynthesis.^84^ The expression of photosynthetic genes is influenced by environmental factors, particularly light, which regulates gene expression through photoreceptors like phytochromes. These photoreceptors coordinate the expression of nuclear and plastid genes, ensuring the proper assembly and function of photosynthetic complexes.^85^^,^^86^ Anterograde signaling from the nucleus to the chloroplast involves nuclear-encoded factors such as sigma factors and RNA-binding proteins, which regulate chloroplast gene expression.^85^ Conversely, retrograde signaling from chloroplasts to the nucleus involves photosynthetic processes that influence nuclear gene expression.^85^ The ubiquitin-proteasome system (UPS) also plays a role in regulating photosynthesis by degrading chloroplast proteins through the CHLORAD pathway, highlighting the interplay between nuclear and promising route towards crop improvementchloroplast systems.^87^ Specific nuclear-encoded proteins, such as plastid ribosome-associated protein 1 (PBR1), regulate the translation of chloroplast genes like hypothetical chloroplast reading frame 1 (Ycf1), which is crucial for the biogenesis of photosynthetic complexes (Yang et al., 2016). Additionally, the nuclear control of plastid gene expression is exemplified by the NCP protein, which links phytochrome signaling to the activation of plastid-encoded genes.^88^ The biogenesis of PSII is another example where nuclear-encoded proteins, such as LPE1, facilitate the translation of chloroplast mRNAs in a light-dependent manner, ensuring efficient photosynthesis.^89^ Furthermore, the transfer of plastid genes to the nuclear genome, as observed in *Passiflora*, demonstrates the evolutionary adaptation of plants to maintain photosynthetic efficiency despite gene loss in plastids.^90^ Overall, the intricate regulation of photosynthetic efficiency in plants is a result of the dynamic interaction between nuclear and chloroplast genes, mediated by various signaling pathways and environmental cues.

### 3.7 Transcriptional control

The transcriptional control of photosynthetic genes in photosynthetic organisms is intricately coordinated with other cellular processes through a complex network of regulatory mechanisms. Light is a primary environmental signal that influences photosynthetic gene expression, primarily through photoreceptors such as phytochromes and cryptochromes, which regulate transcriptional reprogramming by interacting with master regulatory TFs and chromatin remodeling processes.^85^^,^^91^ These TFs, such as elongated hypocotyl 5 (HY5) and phytochrome interactive factors (PIFs), form dynamic modules that respond to light and temperature cues, directly targeting promoter elements to modulate gene expression for photosynthesis and photoprotection.^92^ Additionally, the coordination between nuclear and chloroplast genomes is achieved through anterograde and retrograde signaling pathways, ensuring synchronized expression of photosynthetic genes across cellular compartments.^85^ In cyanobacteria, transcriptional coordination is evident in the co-regulation of photosystems and metabolic processes, highlighting the adaptability of these organisms to environmental changes.^93^ Furthermore, metabolic signals, such as the TCA cycle metabolite *α*-ketoglutarate, influence chromatin structure and transcription by affecting histone methylation, thereby linking metabolic status to gene expression.^94^ The regulatory networks in plants like Arabidopsis and rice exhibit both conservation and divergence, reflecting evolutionary adaptations to different environmental conditions.^95^ In *Rhodobacter sphaeroides*, transcription factors such as FnrL and PrrA regulate photosynthesis and related processes, demonstrating significant cross-talk and combinatorial regulation.^96^ This intricate network of transcriptional regulation is further complemented by the coordination between transcription and mRNA translation, ensuring efficient gene expression in response to changing environmental conditions.^97^ Overall, the transcriptional control of photosynthetic genes is a highly coordinated process involving multiple layers of regulation, integrating environmental signals, metabolic cues, and inter-compartmental communication to optimize photosynthetic efficiency and cellular adaptation.

Environmental factors significantly influence the transcriptional control of photosynthetic efficiency across different plant species by modulating various physiological and molecular processes. Light, as a primary environmental determinant, regulates photosynthetic gene expression through photoreceptors like phytochrome, which affects transcriptional and posttranscriptional levels in both chloroplast and nuclear genomes.^85^^,^^98^ The HY5-PIF regulatory module exemplifies how light and temperature cues are integrated to modulate photosynthetic gene transcription, ensuring optimal photosynthetic performance and growth under varying conditions.^99^ Additionally, environmental factors such as temperature, CO_2_ concentration, and humidity dynamically influence photosynthesis by affecting processes like stomatal conductance and electron transport, which are crucial for efficient photosynthetic induction and reduction of induction loss.^100^ The role of transcription factors such as CBFs and DREBs in regulating plant responses to extreme temperatures and water availability further underscores the importance of environmental modulation in maintaining high photosynthetic performance.^101^ Moreover, the dynamic reorganization of photosynthetic super complexes in response to environmental cues, as observed in *Chlamydomonas reinhardtii*, highlights the adaptability of photosynthetic machinery to changing conditions.^48^ The interaction between light intensity, spectrum, and redox regulation also plays a critical role in adjusting plant metabolism to environmental changes, thereby influencing photosynthetic efficiency.^102^ Furthermore, studies on *Arabidopsis thaliana* under dynamic light conditions reveal that fluctuating light regimes lead to transcriptional adjustments that mitigate photoinhibition and optimize photosynthetic responses.^103^ Collectively, these findings illustrate the complex interplay between environmental factors and transcriptional control mechanisms that govern photosynthetic efficiency, emphasizing the need for integrated models that incorporate these variables to predict plant responses under changing climatic conditions.^104^^,^^105^

### 3.8 Post-Translational regulation

Post-translational modifications (PTMs) play a crucial role in regulating the photosynthetic machinery, enabling plants to adapt to environmental changes and optimize photosynthesis. Phosphorylation of light-harvesting complexes in plant chloroplasts presents a promising avenue for enhancing photosynthetic efficiency and potentially improving crop yields. The dynamic phosphorylation and dephosphorylation of thylakoid proteins, particularly those in light-harvesting complex II (LHCII) and PSII, are crucial for state transitions and maintaining the redox balance of the electron transfer chain, which are essential for optimal photosynthetic performance under varying light conditions.^1^^,^^106^ Specifically, the phosphorylation of LHCII, mediated by the kinase STATE TRANSITION 7 (STN7), is central to state transitions, allowing plants to acclimate to changing light environments by reallocating light-harvesting capacity between photosystems.^107^ This regulatory mechanism can be exploited to improve photosynthetic efficiency, as demonstrated by the concept of Truncated Light-harvesting chlorophyll Antenna size (TLA), which has shown a 25% increase in biomass accumulation in high-density tobacco canopies by reducing excess light absorption and enhancing solar-to-biomass conversion efficiency.^108^ Additionally, engineering the abundance and composition of antenna proteins, such as through the development of pale green phenotypes, can improve light distribution within plant canopies, thereby enhancing photosynthetic efficiency in crowded environments.^55^ Furthermore, the integration of artificial systems, such as quantum dots and carbon dots, with chloroplasts has been shown to significantly enhance photophosphorylation and ATP production, indicating that hybrid systems can further boost photosynthetic efficiency by optimizing light capture and energy conversion.^109^^,^^110^ These strategies, combined with the understanding of phosphorylation's role in photoprotection and nonphotochemical quenching, particularly in monocots like rice, suggest that manipulating phosphorylation pathways could enhance crop resilience to environmental stresses and improve overall productivity.^111^ Thus, leveraging the phosphorylation of light-harvesting complexes offers a multifaceted approach to optimizing photosynthesis and increasing crop yields.

Acetylation and methylation significantly influence the efficiency of light-harvesting complexes in plant chloroplasts by modulating protein function and structural organization. Acetylation, particularly through the action of chloroplast acetyltransferases such as Gcn5-related *N*-acetyltransferase 2 (GNAT2), plays a crucial role in the organization and dynamics of thylakoid structures, which are essential for efficient light harvesting and state transitions between PSI and PS II.^112^^,^^113^ The GNAT family of enzymes, responsible for acetylating proteins, affects the accumulation of chloroplast-related compounds and the organization of thylakoid protein complexes, which are critical for maintaining the balance of excitation energy between PSI and PSII.^113^^,^^114^ This balance is vital for optimizing photosynthetic efficiency and preventing photodamage, as it ensures that light energy is appropriately distributed and utilized within the photosystems.^112^ Methylation, another post-translational modification, impacts chloroplast function by regulating transcriptional efficiency and metabolic activities, although its specific effects on LHC are less well-documented compared to acetylation.^47^ The precise spatial arrangement of protein subunits and binding cofactors in PSI, facilitated by these modifications, is crucial for achieving high quantum efficiency in photosynthesis.^115^ Additionally, the interaction between chlorophyll and carotenoids within the light-harvesting complexes is sensitive to structural perturbations, which can be influenced by acetylation, thereby affecting the overall lifetime and efficiency of the complexes.^116^ These modifications underscore the complex regulatory mechanisms plants employ to optimize photosynthesis under varying environmental conditions, highlighting the importance of acetylation and methylation in maintaining the efficiency of light-harvesting complexes in chloroplasts.

The optimization of redox-based modifications in light-harvesting complexes can indeed improve photosynthetic efficiency in plant chloroplasts under stress conditions. Redox regulation, particularly through thiol switches, plays a crucial role in balancing the redox state, metabolism, and oxidative stress within chloroplasts, which is essential for maintaining photosynthetic efficiency under fluctuating environmental conditions.^117^ The alternative oxidase (AOX) pathway, which regulates cellular reactive oxygen species (ROS) and redox states, has been shown to optimize photosynthesis during osmotic and temperature stress by modulating the malate valve and antioxidative systems, thereby enhancing stress tolerance.^118^ Additionally, the introduction of alternative electron transport sinks, such as flavodoxin, in chloroplasts has been demonstrated to protect photosynthetic activities under drought stress by modulating the chloroplast redox poise, which in turn affects the expression of stress-responsive genes.^119^ Furthermore, the use of optically matched quantum dots to enhance photophosphorylation in chloroplasts exemplifies how artificial modifications can improve the efficiency of light capture and energy conversion, leading to increased photosynthetic output.^110^ These strategies highlight the potential of redox-based modifications and other innovative approaches to enhance photosynthetic efficiency, particularly under stress conditions, by optimizing the light-harvesting and electron transport processes within chloroplasts.

Glycosylation plays a significant role in regulating the structure and function of LHCs in plant chloroplasts, although it is not as extensively studied as other post-translational modifications like phosphorylation. Glycosylation, along with other modifications, impacts the structural dynamics and functional efficiency of LHCs, which are crucial for photosynthesis. These complexes, particularly LHCII, are responsible for capturing light energy and transferring it to the photosystems, primarily PSII and PSI.^120^^,^^121^ The regulation of light-harvesting is essential for balancing energy distribution between PSI and PSII, especially under varying light conditions, to prevent photodamage and optimize photosynthetic efficiency.^47^^,^^122^ While phosphorylation is well-documented in facilitating state transitions and energy redistribution between photosystems,^123^ glycosylation may influence the structural conformation and stability of LHCs, thereby affecting their interaction with other proteins and pigments. This is crucial for maintaining the delicate balance between light absorption and photoprotection, as LHCs can switch between light-harvesting and energy-dissipating states.^124^^,^^125^ The structural integrity and functional dynamics of LHCs are also modulated by interactions with carotenoids, which are bound through weak intermolecular forces and play roles in both light harvesting and photoprotection.^116^^,^^126^ Although the specific mechanisms by which glycosylation affects LHCs are not fully elucidated, it is likely that this modification, along with others, contributes to the fine-tuning of photosynthetic processes by altering protein interactions and structural configurations within the chloroplast.^47^ Further research into glycosylation and its impact on LHCs could provide deeper insights into the regulation of photosynthesis and the adaptation of plants to fluctuating light environments.

## 4 Advances in molecular regulation

Recent advances in the molecular regulation of photosynthesis have significantly enhanced our understanding of plant energy production. These advances span various aspects of photosynthetic processes, including redox regulation, electron transport, post-translational modifications, and chloroplast gene expression. By exploring these areas, researchers have uncovered mechanisms that not only improve photosynthetic efficiency but also offer potential applications in crop improvement and renewable energy production. The following sections detail these key advancements. Redox homeostasis plays a crucial role in maintaining photosynthetic efficiency. ROS act as signaling molecules that regulate the electron transport chain and PSI and PSII, ensuring balanced energy production and photo-protection.^127^ Thiol-based systems and antioxidative enzymes are integral to this regulation, helping to maintain photosynthesis homeostasis and enhance plant productivity under stress conditions.^127^ Recent studies have highlighted the importance of regulatory mechanisms in photosynthetic electron transport, such as state transitions and cyclic electron flow, which help plants acclimate to changing environmental conditions.^128^

Flavodiiron enzymes have been identified as key players in oxygen reduction, contributing to the dissipation of excess energy and protection of the photosynthetic apparatus.^128^ PTMs, including phosphorylation and thioredoxin-mediated redox regulation, are critical for modulating protein function in response to environmental changes. These modifications impact light energy distribution between photosystems and the Calvin cycle's efficiency.^47^ Recent advances have identified additional PTMs, such as acetylation and nitration, which regulate chloroplast functions and photosynthetic efficiency.^47^ Advances in understanding chloroplast gene expression have revealed mechanisms that optimize photosynthesis and minimize photodamage. These include the engineering of pentatricopeptide repeat proteins, which enhance RNA processing and stress tolerance.^84^ However, light-activated gene expression in chloroplasts is crucial for efficient energy conversion and has implications for improving crop yields.^84^ The integration of photosystem complexes with nanostructured electrodes has opened new avenues for renewable energy production, such as hydrogen generation and electricity production in photo-bioelectrochemical cells.^25^ The use of whole-cell microorganisms, like cyanobacteria and microalgae, has shown promise in enhancing photosynthetic energy conversion for sustainable energy solutions.^25^ Photosynthetic super complexes undergo dynamic reorganizations in response to environmental cues, such as light and temperature changes. These reorganizations include qE quenching and cyclic electron flow, which are essential for maintaining photosynthetic efficiency.^48^

While these advances have significantly improved our understanding of photosynthesis, challenges remain in fully harnessing these insights for practical applications. The complexity of photosynthetic regulation and the interplay between various molecular mechanisms require further exploration to optimize photosynthetic efficiency and address global challenges such as food security and climate change. Future research may focus on integrating these molecular insights with biotechnological approaches to enhance crop productivity and develop sustainable energy solutions.

## 5 Implications for crop improvement

Engineering photosynthesis holds significant potential for enhancing crop yields, especially in the face of varying environmental conditions. This approach involves manipulating the photosynthetic process to improve efficiency, which can lead to increased biomass and yield. The implications of such advancements are profound, as they could help meet the growing global food demand and mitigate the impacts of climate change on agriculture. The following sections explore the potential benefits and challenges of photosynthesis engineering in different environmental contexts. Recent studies have demonstrated that genetic manipulation of photosynthesis can lead to substantial yield increases. For instance, improvements in the Calvin cycle and electron transport have shown yield increases of over 40% in controlled environments.^18^ Engineering efforts have focused on enhancing the enzyme-limited (Ac) and electron transport-limited (Aj) rates, with simulations predicting yield gains of up to 8% in optimal conditions.^129^ Bioengineering strategies that accelerate recovery from photoprotection have increased photosynthetic efficiency and seed yield by up to 33% in soybean field trials.^54^ Engineering photosynthesis to be resilient to climate change is crucial. Strategies that perform well at higher temperatures and CO_2_ levels are being developed, which could ensure stable yields under future climate scenarios.^130^^,^^131^ One approach involves the overexpression of specific enzymes and transcription factors associated with photosynthesis, such as the CAM-specific phosphoenolpyruvate carboxylase (PEPC) from *Agave americana*, which has been shown to improve both photosynthetic efficiency and stress tolerance in transgenic tobacco plants. These plants exhibited increased biomass and enhanced tolerance to salt and drought stress, indicating a promising avenue for improving plant resilience through photosynthetic modifications.^132^ Citric acid (CA) application also contributes to stress tolerance by improving photosynthetic rates and reducing reactive oxygen species, further supporting the role of metabolic engineering in stress management.^133^ Moreover, the protection and repair of PSII are crucial, as photoinhibition under abiotic stress can severely impair photosynthesis. Enhancing the repair mechanisms of PSII, such as through the turnover of the D1 protein, can mitigate these effects.^134^ Transcription factors from CAM plants, like those from the NAC family, have been identified as potential genetic engineering targets to improve water-use efficiency and stress tolerance by regulating stress-responsive genes.^135^^,^^136^ Furthermore, the overexpression of transcription factors like ATHB17 in Arabidopsis has been shown to enhance stress tolerance by modulating the expression of photosynthesis-associated nuclear genes and plastid sigma factors, which are crucial for maintaining photosynthetic efficiency under stress.^137^ Lastly, increasing the levels of antioxidants such as ascorbate and carotenoids like astaxanthin can bolster oxidative stress tolerance, providing another layer of protection for the photosynthetic machinery.^138^^,^^139^ Moreover, the development of climate-smart crops, such as those with facultative C_3_-C_4_ metabolism, allows plants to switch between photosynthetic pathways based on environmental conditions, potentially increasing resilience to temperature and CO_2_ fluctuations.^140^ While enhancing photosynthesis can lead to yield improvements, the outcomes are highly dependent on environmental factors such as water and nitrogen availability. Enhancements in Ac alone provide consistent but smaller gains, whereas Aj enhancements offer larger gains but are less effective in marginal environments.^129^

The interaction between photosynthesis and other plant processes, such as stomatal conductance and carbon allocation, needs further exploration to optimize yield outcomes across diverse environments.^2^^,^^141^ The potential for photosynthesis engineering to contribute to food security is significant, but it requires a concerted effort in research and development. This includes exploring the genomic potential of model plants and expanding the scope of engineering efforts to include traits that address the yield gap.^8^^,^^140^ Despite the promise of photosynthesis engineering, traditional breeding and crop management practices remain essential. A combination of these approaches, supported by increased investment and regulatory adjustments, will be necessary to achieve sustainable yield improvements.^131^ While engineering photosynthesis presents a promising avenue for increasing crop yields, it is not without challenges. The success of these efforts will depend on a comprehensive understanding of plant-environment interactions and the integration of multiple strategies to address the complex dynamics of crop growth and yield formation. As research progresses, the potential for photosynthesis engineering to contribute to global food security becomes increasingly apparent, but it must be pursued alongside traditional agricultural practices to ensure a holistic approach to crop improvement. Together, these studies underscore the multifaceted strategies available for engineering photosynthesis to enhance abiotic stress tolerance, offering promising solutions for improving crop resilience in the face of climate change.

## 6 Insights from structural biology and biophysics of photosystems

Insights from the structural biology and biophysics of photosystems are invaluable for designing more efficient artificial photosynthetic systems, as they provide a detailed understanding of natural photosynthetic processes and components. The structural and functional principles of natural photosystems, such as PSII and PSI, serve as blueprints for artificial systems. For example, the complex biochemical architecture of PSII, particularly the oxygen-evolving manganese–calcium cluster, is critical for water splitting and can guide the development of effective catalysts in synthetic systems.^142^ Similarly, the high quantum efficiency of PSI, attributed to its precise spatial arrangement of protein subunits and cofactors, can guide the development of artificial systems with optimized light-harvesting capabilities.^115^ The structural variability and adaptability of photosynthetic machinery, such as the formation of IsiA-PSI super complexes in cyanobacteria, highlight the importance of organizational flexibility in enhancing light absorption and electron transport, which can be mimicked in artificial systems.^143^ Furthermore, the principles of proton-coupled electron transfer (PCET) in natural photosynthesis, which link redox processes with proton gradients, are essential for designing artificial systems that efficiently convert solar energy into chemical energy.^144^ Advances in material science, inspired by the natural photosystems, emphasize the integration of photosystems as functional materials in artificial constructs, suggesting that reengineering these features can meet human energy demands.^145^ Additionally, the development of biohybrid systems, which expand light-harvesting capabilities through the conjugation of natural and artificial chromophores, demonstrates the potential for enhancing energy transfer efficiency beyond traditional theories like Förster resonance energy transfer (FRET).^146^ These insights collectively underscore the potential of leveraging structural biology and biophysics to create more efficient and sustainable artificial photosynthetic systems, capable of addressing global energy challenges.^147^^,^^148^

## 7 Technological innovations in photosynthesis research

Technological innovations in photosynthesis research hold significant potential for addressing global food and energy security challenges by enhancing crop productivity and developing sustainable energy sources. Increasing the efficiency of photosynthesis in crop plants is crucial to meet the rising global food demand driven by population growth and biofuel mandates. This can be achieved through genetic manipulation and synthetic biology to improve photosynthetic efficiency and performance, which could lead to substantial increases in crop yields and bioenergy production.^2^^,^^149^ Key areas for improvement include stomatal and mesophyll conductance, biochemical capacity, and carbon fixation pathways, which are essential for adapting photosynthesis to environmental changes such as light intensity, temperature, and elevated CO_2_ levels.^2^ Furthermore, photosynthesis research is pivotal in developing clean energy systems, such as solar fuels and biohydrogen, which are vital for reducing reliance on fossil fuels and mitigating climate change impacts.^150^ The intricate process of oxygenic photosynthesis, which efficiently converts solar energy into chemical energy, serves as a model for sustainable energy solutions and inspires innovations in agriculture and environmental protection.^151^ Despite the challenges posed by the deeply rooted genetic controls of photosynthesis, advancements in monitoring and modeling techniques, such as the European Space Agency's Fluorescence Explorer mission, offer new opportunities to enhance photosynthetic capacity and crop yields, thereby contributing to global food security.^152^

### 7.1 High-throughput phenotyping and imaging technologies

High-throughput phenotyping and imaging technologies have significantly advanced the study of photosynthesis dynamics across various plant species by enabling detailed, non-destructive, and large-scale analysis of photosynthetic traits. The development of platforms like the dynamic environmental photosynthesis imager (DEPI) allows for continuous and high-throughput measurements of photosynthetic parameters under dynamic environmental conditions, revealing emergent phenotypes that are not observable under static laboratory conditions.^153^ Similarly, chlorophyll fluorescence imaging has been utilized to phenotype dynamic photosynthesis and photoprotection in leaves, providing insights into cultivar-specific responses to light regimes and enabling the rapid screening of large plant populations.^154^ The integration of phenomics and genomics is crucial for dissecting the genetic architecture of photosynthesis, with high-throughput phenotyping facilities designed to accommodate fluctuating environmental conditions, thereby aiding in the identification of genetic variations that enhance photosynthetic efficiency (van.^155^ Automated platforms like the Phenovator facilitate the screening of thousands of plants for photosynthetic traits, allowing for the observation of genetic differences and temporal fluctuations in heritability, which are critical for breeding programs.^156^ Hyperspectral imaging, particularly at the canopy level, provides a promising approach to characterizing photosynthetic activities, with systems like GPhenoVision enabling the estimation of solar-induced fluorescence (SIF) and photosynthetic efficiency, which are essential for breeding and genetic studies.^91^ The use of SIF yield, rather than SIF alone, has shown a stronger correlation with photosynthetic capacity, offering a robust method for field phenotyping.^157^ These technologies, combined with advanced modeling approaches, allow for the optimization of canopy photosynthesis and resource use efficiencies, which are pivotal for improving crop yields under varying environmental conditions.^158^ Overall, the integration of high-throughput phenotyping and imaging technologies with genetic and environmental data is transforming our understanding of photosynthesis, providing new opportunities for enhancing plant productivity and resilience.^159^

### 7.2 Computational modeling of photosynthetic processes

Computational models of photosynthetic processes significantly enhance our understanding of plant physiology by integrating complex biochemical and biophysical interactions into predictive frameworks. These models allow researchers to simulate and analyze the intricate dynamics of photosynthesis under various environmental conditions, thereby providing insights into plant metabolic pathways and their regulation. For instance, the integration of chlorophyll fluorescence parameters with genome-scale metabolic models, as demonstrated in the study of the diatom *Phaeodactylum tricornutum*, improves the predictive accuracy of growth rates and metabolic pathway usage, revealing strategies for photoacclimation and potential biotechnological applications.^54^ Similarly, models based on the Farquhar–von Caemmerer–Berry framework have been instrumental in understanding CO_2_ assimilation and the spatial heterogeneity of photosynthesis under stress conditions like excess light and drought.^160^ These models are crucial for exploring the effects of environmental stressors on photosynthetic efficiency and resource allocation, as seen in C_3_ and C_4_ plants, where evolutionary history influences current physiological responses.^161^ Moreover, advancements in sun-induced chlorophyll fluorescence (SIF) modeling provide a novel approach to estimate photosynthetic traits such as the maximum carboxylation rate and stomatal conductance, enhancing the accuracy of physiological predictions and offering new avenues for remote sensing applications.^162^^,^^163^ The development of dynamic models that account for fluctuating light conditions further refines our understanding of photosynthetic responses, highlighting the importance of non-steady-state conditions in plant growth and productivity.^4^ Overall, these computational models bridge the gap between molecular-level processes and ecosystem-scale phenomena, offering valuable tools for predicting plant responses to climate change and guiding efforts in crop improvement and sustainable agriculture.^164^^,^^165^

### 7.3 Omics approaches (genomics, transcriptomics, proteomics) in identifying key genes and pathways

**Figure 5.**
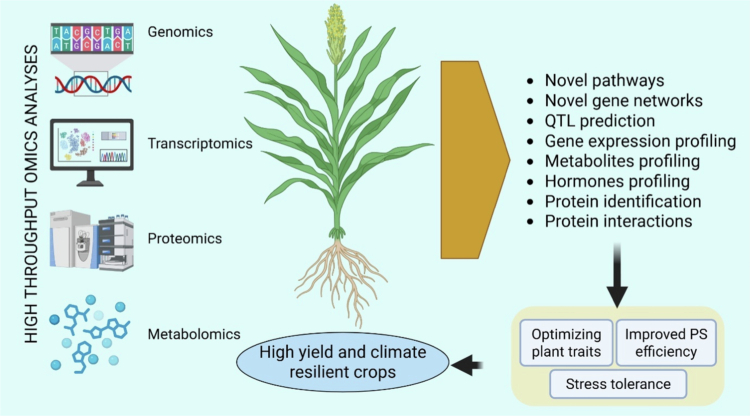
Omics approaches (genomics, transcriptomics, proteomics, and metabolomics) in identifying key genes and pathways: These omics platforms facilitate the discovery of novel pathways and gene networks, QTL prediction, comprehensive gene and metabolite profiling, hormonal analysis, protein identification, and interaction mapping. Collectively, these datasets support the development of high-yielding and climate-resilient crops by enabling the optimization of key plant traits, improvement of photosynthetic efficiency, and enhancement of stress tolerance mechanisms.

Omics-driven research holds significant potential for improving crop yields and photosynthetic efficiency by leveraging advanced technologies such as genomics, transcriptomics, proteomics, and metabolomics. These approaches enable a comprehensive understanding of the genetic and molecular bases of complex traits, facilitating the development of high-yield and climate-resilient crops (Figure 5). For instance, multi-omics integration can elucidate the relationships between crop genomes and phenotypes, allowing for the prediction and enhancement of traits under specific environmental conditions.^166^^,^^167^ The integration of omics data with systems biology provides a holistic understanding of the dynamic interactions within plant systems, enabling the modeling and prediction of cellular functions that underpin yield and stress tolerance.^168^ Moreover, optimizing plant developmental traits, such as leaf structure and vascular architecture, can significantly impact photosynthetic efficiency and overall crop performance.^169^ The application of omics technologies also extends to understanding and enhancing the plant's response to abiotic stresses, which is vital for maintaining productivity in changing climates.^170^^,^^171^ More recently, integrative omics coupled with phenomics and machine learning has revolutionized the identification of candidate genes and pathways associated with photosynthetic performance and yield stability. For example, a recent study by Amin et al.^172^ demonstrated that combining genomics, transcriptomics, and digital phenotyping improved genotype-to-phenotype predictions in wheat under heat stress, enhancing selection accuracy for resilient cultivars. Similarly, Duan et al.^173^ applied metabolomics and proteomics integration to rice to uncover regulatory networks controlling chlorophyll biosynthesis and light-use efficiency, leading to targeted bioengineering of chloroplast function. Furthermore, single-cell multi-omics approaches now enable dissection of cell-specific regulatory mechanisms of photosynthesis and carbon metabolism,^174^ revealing spatial heterogeneity in mesophyll and bundle-sheath cells that could be exploited for engineering improved carbon fixation efficiency. The convergence of omics with CRISPR-based genome editing has also accelerated trait improvement; integrating transcriptome-wide association studies with genome-editing targets has successfully modified photosynthetic enzymes and stress-response genes in soybean and maize.^175^^,^^176^ Such integrative frameworks are evolving toward predictive, data-driven breeding pipelines, termed next-generation omics breeding that combine genomic selection, environmental modeling, and phenomics to inform crop design under future climate scenarios.^177^^,^^178^ Despite ongoing challenges, such as data harmonization, high computational demands, and limited field-scale validation, these advances underscore the transformative power of omics in developing climate-resilient, high-yield crops.^179^ Future directions emphasize integrating multi-omics with artificial intelligence, remote sensing, and high-throughput phenotyping to accelerate breeding cycles and improve real-time monitoring of photosynthetic dynamics at the canopy level.^180^

## 8 Genetic and biotechnological approaches to enhance photosynthetic efficiency

Genetic engineering offers promising avenues to enhance photosynthetic efficiency in crops, which is crucial for meeting the increasing global food demand. One approach involves optimizing the Calvin cycle and electron transport processes, which have shown potential to increase crop yields by over 40% in both controlled and field environments.^18^ Another strategy focuses on exploiting natural genetic variation in photosynthesis, which remains largely untapped due to the complexity of the trait involving thousands of genes. Advances in phenotyping and genetic tools are beginning to uncover quantitative trait loci that could be used in breeding programs to enhance photosynthetic traits.^181^ In cereal crops, where traditional yield improvements have plateaued, genetic diversity in photosynthesis is being explored using high-throughput techniques and low-cost genotyping, offering a feasible path to improve radiation use efficiency and biomass production.^182^ Additionally, engineering C_4_ photosynthetic traits into C_3_ crops is being pursued to enhance productivity, although this requires a deep understanding of the metabolic differences between these pathways.^36^ A significant focus has been on RuBisCO, the enzyme responsible for CO_2_ fixation, where engineering efforts have introduced more efficient versions from cyanobacteria into plants like tobacco, paving the way for similar enhancements in crop species.^183^^,^^184^ The integration of cyanobacterial CO_2_-concentrating mechanisms (CCM) into C_3_ crops like wheat and rice is another promising strategy, with potential improvements in photosynthesis by up to 28%.^185^ Despite these advances, challenges remain, such as optimizing stomatal and mesophyll conductance and adapting photosynthetic processes to environmental changes.^2^ The introduction of novel genetic diversity, including photosynthetic variants from algae and synthetic platforms like cyanobacteria, could further enhance photosynthetic efficiency, although these approaches are still in developmental stages.^186^ Overall, genetic engineering, combined with conventional breeding, holds significant potential to improve photosynthetic efficiency and crop yields, but requires substantial investment and regulatory support to transition from laboratory successes to field applications.^131^

### 8.1 CRISPR/Cas9 and gene editing for photosynthetic traits

**Figure 6.**
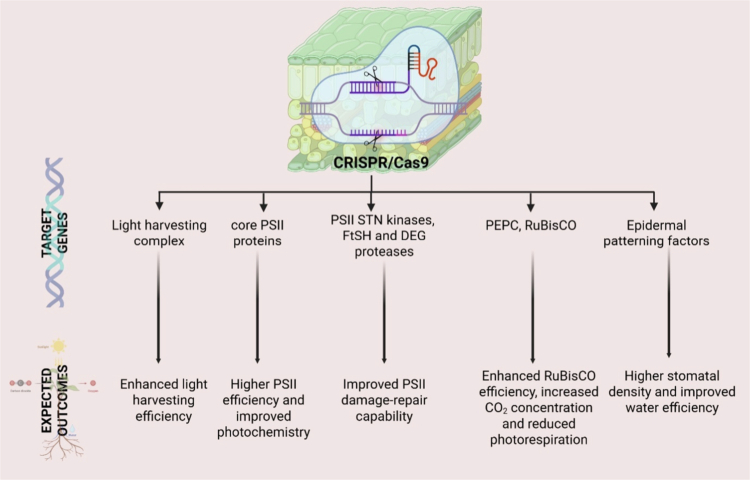
CRISPR/Cas9-mediated optimization of photosynthetic performance in plants: This figure shows CRISPR/Cas9 gene-editing targets to improve plant photosynthesis and water use in plants. It focuses on genes for light-harvesting complexes, PSII proteins, STN kinases, FtSH and DEG proteases, PEPC, RuBisCO, and epidermal patterning. Editing these genes leads to enhanced light harvesting efficiency, improved PSII photochemistry and damage repair, increased RuBisCO efficiency with reduced photorespiration, and higher stomatal density resulting in better water use efficiency. Together, these changes boost photosynthetic efficiency and water use, promoting better crop productivity and stress tolerance.

The CRISPR/Cas9 system has significantly advanced the field of gene editing, particularly in enhancing photosynthetic traits in crops, which is crucial for improving agricultural productivity and sustainability. This technology allows for precise modifications of plant genomes, enabling the enhancement of photosynthetic efficiency and yield in crops such as rice and tomatoes (Figure 6). For instance, the knockout of the hexokinase gene *OsHXK1* in rice using CRISPR/Cas9 has led to increased photosynthetic efficiency and higher yields, demonstrating the potential of this technology in optimizing photosynthetic traits for better crop performance.^187^ Similarly, CRISPR/Cas9 has been employed in tomato breeding to improve various traits, including those related to photosynthesis, by facilitating the introgression of elite traits from wild relatives into cultivated varieties.^188^ The versatility of CRISPR/Cas9 is further highlighted by its ability to perform multiplex gene editing, allowing simultaneous modifications of multiple genes, which is particularly beneficial for complex traits like photosynthesis that involve multiple genetic pathways.^189^^,^^190^ Advances in CRISPR/Cas9 technology, such as base and prime editing, have expanded the toolkit available for precise genetic modifications, enabling the creation of site-specific mutations that can enhance photosynthetic efficiency.^191^^,^^192^ Moreover, the development of new delivery methods, including nanotechnology and viral vectors, has improved the efficiency of CRISPR/Cas9 applications in plants, overcoming traditional bottlenecks associated with gene editing in plant systems.^190^^,^^192^ These technological advancements underscore the transformative potential of CRISPR/Cas9 in improving photosynthetic traits, thereby contributing to the development of crops that are more resilient to environmental stresses and capable of meeting the growing global food demand.^193^^,^^194^ As CRISPR/Cas9 continues to evolve, its application in enhancing photosynthetic traits will likely play a pivotal role in future agricultural innovations.

### 8.2 Synthetic biology for optimizing light utilization and energy conversion

Synthetic biology offers innovative strategies to optimize light utilization and energy conversion, particularly in enhancing photosynthesis and developing biohybrid systems. One approach involves engineering plant carbon fixation processes to improve photosynthetic efficiency. This includes modifying the enzyme RuBisCO for better activity and specificity, altering enzyme expression in the Calvin cycle, and introducing carbon-concentrating mechanisms like C_4_ metabolism to enhance biomass production.^195^ Additionally, synthetic biology has been employed to create polychromatic solar energy conversion systems by combining photosynthetic proteins from different organisms, such as recombinant plant light-harvesting complexes with bacterial reaction centers, to enhance light absorption and energy conversion capabilities.^196^ Light-driven synthetic systems also play a crucial role in controlling biological processes with high precision. Optogenetics, for instance, uses light to regulate gene expression and protein interactions, offering spatiotemporal control over cellular activities.^197^^,^^198^ In the realm of artificial photosynthesis, biohybrid systems integrate biological specificity with synthetic nanomaterials to facilitate solar-to-chemical conversion. These systems leverage the efficient light absorption and charge separation properties of nanomaterials, coupled with biological catalysts, to drive redox reactions and enhance energy conversion efficiency.^199^ Furthermore, engineering living photovoltaics involves creating interfaces between living cells and electron-accepting scaffolds to improve electron transport and solar conversion efficiency, with engineered photosynthetic organisms showing potential for biophotovoltaic applications.^200^ These diverse synthetic biology approaches collectively aim to optimize light utilization and energy conversion, addressing the growing demand for sustainable energy solutions and increased agricultural productivity.

## 9 Integrating photosynthetic efficiency with stress tolerance

Integrating photosynthetic efficiency with stress tolerance is crucial for enhancing plant productivity under changing environmental conditions. By improving the efficiency of light capture, carbon fixation, and energy utilization, plants can maintain higher growth rates even under drought, salinity, or temperature stress. Advances in molecular breeding and genetic engineering allow for simultaneous optimization of photosynthetic pathways and stress-responsive mechanisms. This integration supports better water-use efficiency, reduced photooxidative damage, and improved resilience. Ultimately, linking these traits provides a sustainable approach to developing climate-smart crops capable of maintaining yield stability in adverse environments.

### 9.1 Photosynthesis under abiotic stresses (drought, heat, salinity)

Abiotic stresses such as drought, salinity, and extreme temperatures profoundly impair the photosynthetic machinery of plants by disrupting key physiological and biochemical pathways. These stresses collectively reduce photosynthetic efficiency, an essential determinant of plant growth and productivity, by affecting chlorophyll biosynthesis, photosystem integrity, electron transport, and gas exchange processes.^1^ Understanding these stress-induced perturbations is vital for devising strategies to enhance plant resilience under adverse conditions. However, drought stress primarily impedes photosynthesis through stomatal closure, which limits CO₂ assimilation and decreases photosynthetic rates. This restriction leads to chlorophyll degradation, disruption of the electron transport chain, and diminished efficiency of PS I and II.^201^^,^^202^ Additionally, drought-induced oxidative stress damages cellular structures, while antioxidant defense mechanisms are activated to mitigate such damage.^203^ The concurrent reduction in leaf area and stem elongation further constrains light interception and photosynthetic capacity.^203^ Moreover, salinity stress similarly reduces photosynthetic efficiency by inducing ionic imbalances, particularly via Na⁺ accumulation, which disrupts cellular homeostasis and damages chloroplast ultrastructure.^204^^,^^205^ The resulting osmotic stress limits water uptake and triggers stomatal closure, thereby restricting CO₂ availability.^201^^,^^204^^,^^206^ Also, salinity interferes with the biosynthesis of photosynthetic pigments and Calvin cycle enzymes, exacerbating the decline in photosynthetic performance.^207-209^ Furthermore, extreme temperatures, both high and low, further compromise photosynthetic processes.^210^^,^^211^ Elevated temperatures increase photorespiration and destabilize thylakoid membranes, leading to reduced carbon fixation efficiency.^212^ Conversely, cold stress decreases membrane fluidity and impairs photosystem function, thereby limiting light absorption and energy conversion.^213^^,^^214^ Plants have evolved diverse adaptive mechanisms to counter these stress effects, including the accumulation of osmoprotectants such as glycine betaine, which stabilizes proteins and membranes.^215^ Stress hormones, notably ABA, mediate stomatal regulation and activate downstream defense pathways.^216^ Furthermore, genetic and biochemical adaptations, such as the expression of stress-responsive genes and proteins, help sustain photosynthetic activity under stress conditions.^208^^,^^217^ Notably, some species demonstrate inherent resilience; for example, thermophilic plants like *Ziziphus spina-christi* exhibit enhanced photosynthetic performance under elevated temperatures.^214^ Understanding the complex interplay among multiple abiotic stresses and their cumulative effects on photosynthesis is essential for developing climate-resilient, high-performing crop varieties.^204^

#### 9.1.1 Synergistic improvement of photosynthesis and stress resilience

The synergistic improvement of photosynthesis and stress resilience in plants is underpinned by a complex interplay of genetic and molecular mechanisms. One promising strategy to simultaneously enhance photosynthetic efficiency and stress tolerance involves the overexpression of key regulatory genes. For example, the ectopic expression of a CAM-specific gene from *Agave americana* in tobacco significantly improved photosynthetic rates and biomass accumulation. Moreover, this genetic modification enhanced salt and drought tolerance by promoting proline biosynthesis and modulating carbon metabolism, thereby strengthening the plant’s overall physiological resilience.^132^ Similarly, the introduction of a pyruvate-phosphate dikinase gene from *Suaeda monoica* into C_3_ plants has been demonstrated to enhance photosynthesis by reducing photorespiration and improving stress tolerance under elevated CO_2_ conditions.^218^ Additionally, the manipulation of chloroplast redox pathways, which are crucial for photosynthetic electron transport and CO_2_ assimilation, has been shown to increase stress tolerance and plant growth by modulating redox signals that influence plant responses to environmental stresses.^219^ The regulation of photosynthesis-associated nuclear genes and plastid-encoded genes by transcription factors such as ATHB17 also plays a significant role in enhancing stress tolerance by adjusting gene expression in response to abiotic stresses.^137^ Furthermore, the integration of these genetic and molecular strategies, including the modulation of photorespiration, cyclic electron flow, and alternative oxidase pathways, optimizes photosynthesis and provides a robust defense against abiotic stress.^220^ Collectively, these findings highlight the potential of genetic engineering and molecular interventions to simultaneously enhance photosynthetic efficiency and stress resilience in plants, offering promising avenues for improving crop productivity in the face of climate change.^2^

## 10 Challenges and limitations

Engineering photosynthesis for crop improvement confronts complex biological, technical, and translational barriers. Converting C₃ crops to a C₄-like physiology requires extensive reprogramming of regulatory networks and induction of Kranz anatomy, yet progress remains limited by an incomplete systems-level understanding.^39^ Efforts to transplant algal or cyanobacterial CO₂-concentrating mechanisms into C₃ plants necessitate coordinated reconstruction of carboxysomes, transporters, and compatible metabolic fluxes.^221^ Similarly, the introduction of CAM metabolism depends on decoding circadian regulation and achieving stable multigene integration.^222^ Even when biochemical constraints such as stomatal conductance or Rubisco kinetics are alleviated, yield gains are often modest, revealing canopy-scale limitations.^2^^,^^179^ As climate change alters temperature, vapor pressure deficit, and radiation regimes, engineered traits must also demonstrate resilience under variable field conditions.^130^ Moreover, maximizing photosynthetic efficiency while improving abiotic stress tolerance involves inherent trade-offs. Drought, salinity, and heat can suppress chlorophyll biosynthesis, damage photosystems, and impair electron transport, thereby constraining productivity.^201^ Increases in photosynthetic rate do not always translate into higher yield, underscoring the importance of optimizing canopy light distribution and source–sink balance.^179^ Stress-tolerant genotypes often incur growth–defense penalties as resources are redirected from biomass accumulation to protection.^223^ Light quality and intensity can act as both stimuli and stressors, and the complex regulation of auxiliary pathways, photorespiration, cyclic electron flow, and alternative oxidase activity remains poorly characterized under fluctuating environments.^220^ Transgenic strategies that modify these circuits must therefore account for potential trade-offs and environmental contingencies.^224^ Achieving durable productivity and resilience requires multidisciplinary approaches and attention to trait plasticity across ecological gradients.^225^^,^^226^

Beyond these biochemical and anatomical challenges, integrating multi-omics data represents a new frontier but also introduces computational and translational limitations. Combining genomics, transcriptomics, proteomics, and metabolomics enables systems-level insights, yet inconsistencies in data quality, normalization, and cross-platform compatibility hinder robust integration.^172^ Predicting field-relevant phenotypes from omics data requires advanced statistical and machine-learning frameworks capable of capturing non-linear gene-environment interactions. Furthermore, the high cost and complexity of data acquisition constrain routine use in breeding programs. Accurate translation of omics findings to field performance also depends on high-throughput phenotyping. Remote sensing and imaging platforms can monitor photosynthetic efficiency, chlorophyll fluorescence, and canopy temperature at scale, but linking these data to genetic markers remains challenging. Environmental variability, including fluctuating CO₂, temperature, and radiation, can alter gene expression and metabolite fluxes, reducing model transferability across sites and seasons. These uncertainties emphasize the need for robust environmental modeling and multi-location trials. Moreover, genome editing and transformation efficiency further limit progress. Despite advances in CRISPR/Cas technologies, genotype-dependent regeneration and off-target mutations remain barriers in several major crops.^227^ Complex photosynthetic traits often require precise manipulation of multiple genes, demanding innovations in promoter design, gene stacking, and spatial regulation. Field validation under realistic climate scenarios is essential to assess the stability and scalability of engineered traits. In summary, photosynthesis engineering is not a single breakthrough but a continuum of innovations, from systems-level discovery to precise editing, scalable phenotyping, and context-specific validation.

## 11 Future Directions and Implications for Agriculture

Genetic engineering has demonstrated notable progress in this area, with alterations to the Calvin cycle and electron transport chains achieving yield increases of over 40% in certain cases.^18^ A major focus of this research lies in improving RuBisCO’s carboxylation efficiency, a key determinant of CO₂ fixation and biomass accumulation.^183^ Additionally, the incorporation of CO₂-concentrating mechanisms from other organisms and enhancements in photoprotection recovery have led to higher photosynthetic rates and up to 33% greater seed yields without compromising quality.^54^ As conventional breeding approaches approach physiological yield limits, targeted metabolic engineering emerges as a crucial strategy to maintain productivity in response to growing global demands.^8^ Moreover, cross-scale modeling suggests that enhancing both enzyme-limited and electron transport-limited phases of photosynthesis can yield substantial productivity gains across diverse environments.^2^ In C_4_ crops, efforts to elevate RuBisCO content and electron transport capacity show potential for sustaining yields under fluctuating conditions.^5^ The integration of such genetic and biochemical innovations into climate-smart crop designs can contribute to sustainable intensification while mitigating environmental impacts.^140^^,^^149^^,^^152^

Despite these advances, translating molecular improvements into tangible field-level outcomes remains challenging. Yield depends on canopy-scale photosynthesis over time rather than instantaneous leaf activity, leading to molecular-level gains to produce only modest yield increases of 10–15% in many cases.^179^ Moreover, the inherent inefficiency of photosynthesis and its sensitivity to fluctuating light, temperature, and water conditions constrain its effectiveness in natural environments.^82^^,^^228^ To bridge this gap, holistic models that incorporate canopy dynamics, temporal variability, and overlooked components such as non-foliar photosynthesis are essential.^18^ Alongside biological optimization, socio-economic considerations, including product profiling, cost–benefit assessments, and market acceptability, must guide the implementation of new technologies.^229^ Artificial photosynthesis, while conceptually promising for sustainable carbohydrate synthesis, is still constrained by the thermodynamic stability of CO₂ and the complexity of replicating biological efficiency.^230^ Therefore, multidisciplinary collaboration across molecular biology, agronomy, and socio-economic fields is crucial to realize the practical benefits of enhanced photosynthesis.^6^

Climate change adds further complexity to agricultural productivity. While elevated CO₂ may benefit crops in cooler regions, yields in tropical zones, particularly rice and maize are projected to decline.^231^ Global analyzes already indicate yield reductions of up to 13.4% for oil palm and minimal gains for soybean, resulting in net calorie losses.^232^ Increasing droughts and heatwaves further exacerbate risks to maize, wheat, and rice production.^233^^,^^234^ Economic assessments reveal that such yield variability could reduce GDP and welfare in vulnerable regions.^235^ Projections for the 2050s suggest generally negative effects on staple crops under warming scenarios, highlighting the urgency of adaptive measures.^236^^,^^237^ Consequently, integrating photosynthetic innovations with climate-smart agricultural practices is essential for bolstering global food security and mitigating climate change.^129^^,^^238^

## 12 Conclusion

Recent advances in photosynthesis research are transforming strategies for improving crop productivity and sustainability. Innovations in synthetic biology, nanotechnology, and molecular engineering now enable precise optimization of light reactions, Calvin cycle enzymes, and CO₂-concentrating mechanisms in C₃ plants, offering substantial potential for yield enhancement under climate stress. The development of climate-smart crops capable of modulating between C₃ and C₄ metabolism exemplifies the integration of physiological flexibility with efficient carbon utilization. Genetic interventions, including cyanobacterial RuBisCO incorporation and regulatory network redesign, further highlight the feasibility of improving photosynthesis at the molecular level. Complementary progress in mechanistic modeling, omics integration, and remote sensing facilitates fine-tuning of photosynthetic performance from chloroplast to canopy scales. Realizing these innovations will require interdisciplinary collaboration across biology, modeling, and agronomy, alongside policies that balance productivity with ecological sustainability. Together, these advances herald a new era in photosynthesis-driven agricultural resilience and global food security.

## References

[1] Gururani MA, Venkatesh J, Tran LSP. Regulation of photosynthesis during abiotic stress-induced photoinhibition. Mol Plant. 2015b;8:1304–1320. doi: 10.1016/j.molp.2015.05.005. 25997389

[2] Hussain S, Ulhassan Z, Brestic M, Zivcak M, Zhou W, Allakhverdiev SI, Yang X, Safdar ME, Yang W, Liu W. Photosynthesis research under climate change. Photosynth Res. 2021;150:5–19. doi: 10.1007/s11120-021-00861-z. 34235625

[3] Allakhverdiev SI. Optimising photosynthesis for environmental fitness. Funct Plant Biol. 2020;47:iii–vii. doi: 10.1071/FPv47n11_FO. 33046183

[4] Long SP, Taylor SH, Burgess SJ, Carmo-Silva E, Lawson T, Souza AP, De, Leonelli L, Wang Y. Into the shadows and back into sunlight: photosynthesis in fluctuating light. Annu Rev Plant Biol. 2022;73:617–648. doi: 10.1146/annurev-arplant-070221-024745. 35595290

[5] Sales CRG, Wang Y, Evers JB, Kromdijk J. Improving C4 photosynthesis to increase productivity under optimal and suboptimal conditions. J Exp Bot. 2021;72:5942–5960. doi: 10.1093/jxb/erab327. 34268575 PMC8411859

[6] Nuccio ML, Potter L, Stiegelmeyer SM, Curley J, Cohn J, Wittich PE, Tan X, Davis J, Ni J, Trullinger J. Strategies and tools to improve crop productivity by targeting photosynthesis. Philos Trans R Soc B Biol Sci. 2017;372:20160377. doi: 10.1098/rstb.2016.0377. PMC5566877 28808096

[7] Garcia A, Gaju O, Bowerman AF, Buck SA, Evans JR, Furbank RT, Gilliham M, Millar AH, Pogson BJ, Reynolds MP. Enhancing crop yields through improvements in the efficiency of photosynthesis and respiration. New Phytol. 2023;237:60–77. doi: 10.1111/nph.18545. 36251512 PMC10100352

[8] Orr DJ, Pereira AM, Fonseca Pereira P, da, Pereira-Lima ÍA, Zsögön A, Araújo WL. Engineering photosynthesis: progress and perspectives. F1000Research. 2017;6:1891. doi: 10.12688/f1000research.12181.1. 29263782 PMC5658708

[9] Dillard HR. Global food and nutrition security: from challenges to solutions: report of the international Congress of plant pathology 2018, Boston, USA, 29th July–3rd August 2018 with the title plant health in a global economy. Food Secur. 2019;11:249–252.

[10] Ghosh RK, Otto IM, Rommel J. Food security, agricultural productivity, and the environment: economic, sustainability, and policy perspectives. Front Environ Sci. 2022;10:916272. doi: 10.3389/fenvs.2022.916272.

[11] Albahri G, Alyamani AA, Badran A, Hijazi A, Nasser M, Maresca M, Baydoun E. Enhancing essential grains yield for sustainable food security and bio-safe agriculture through latest innovative approaches. Agronomy. 2023;13:1709. doi: 10.3390/agronomy13071709.

[12] Lal R. Food security in a changing climate. Ecohydrol Hydrobiol. 2013;13:8–21. doi: 10.1016/j.ecohyd.2013.03.006.

[13] Grote U. Can we improve global food security? A socio-economic and political perspective. Food Secur. 2014;6:187–200. doi: 10.1007/s12571-013-0321-5.

[14] McCarthy U, Uysal I, Badia-Melis R, Mercier S, O’Donnell C, Ktenioudaki A. Global food security–Issues, challenges and technological solutions. Trends Food Sci Technol. 2018;77:11–20. doi: 10.1016/j.tifs.2018.05.002.

[15] Fears R, Meulen VT, von Braun J. Global food and nutrition security needs more and new science. Sci Adv. 2019;5 eaba2946. 31853503 10.1126/sciadv.aba2946 PMC6910831

[16] Barrett CB. Overcoming global food security challenges through science and solidarity. Am J Agric Econ. 2021;103:422–447. doi: 10.1111/ajae.12160.

[17] Calicioglu O, Flammini A, Bracco S, Bellù L, Sims R. The future challenges of food and agriculture: an integrated analysis of trends and solutions. Sustainability. 2019;11:222. doi: 10.3390/su11010222.

[18] Simkin AJ, Faralli M, Ramamoorthy S, Lawson T. Photosynthesis in non‐foliar tissues: implications for yield. Plant J. 2020;101:1001–1015. doi: 10.1111/tpj.14633. 31802560 PMC7064926

[19] Mirkovic T, Ostroumov EE, Anna JM, van Grondelle R, Govindjee, Scholes GD. Light absorption and energy transfer in the antenna complexes of photosynthetic organisms. Chem Rev. 2017;117(2):249–293. doi: 10.1021/acs.chemrev.6b00002. 27428615

[20] Nelson N, Junge W. Structure and energy transfer in photosystems of oxygenic photosynthesis. Annu Rev Biochem. 2015;84:659–683. doi: 10.1146/annurev-biochem-092914-041942. 25747397

[21] Lundgren MR. C2 photosynthesis: a promising route towards crop improvement? New Phytol. 2020;228:1734–1740. doi: 10.1111/nph.16494. 32080851

[22] Lawson T, Milliken AL. Photosynthesis–beyond the leaf. New Phytol. 2023;238:55–61. doi: 10.1111/nph.18671. 36509710 PMC10953325

[23] Matthews ML. Engineering photosynthesis, nature’s carbon capture machine. PLoS Biol. 2023;21:e3002183. 37432955 10.1371/journal.pbio.3002183 PMC10335658

[24] Prajapati A, Singh MR. Assessment of artificial photosynthetic systems for integrated carbon capture and conversion. ACS Sustain Chem Eng. 2019;7:5993–6003. doi: 10.1021/acssuschemeng.8b04969.

[25] Sekar N, Ramasamy RP. Recent advances in photosynthetic energy conversion. J Photochem Photobiol C Photochem Rev. 2015;22:19–33. doi: 10.1016/j.jphotochemrev.2014.09.004.

[26] Bräutigam A, Gowik U. Photorespiration connects C3 and C4 photosynthesis. J Exp Bot. 2016;67:2953–2962. doi: 10.1093/jxb/erw056. 26912798

[27] Sharwood RE, Sonawane BV, Ghannoum O, Whitney SM. Improved analysis of C4 and C3 photosynthesis via refined in vitro assays of their carbon fixation biochemistry. J Exp Bot. 2016;67:3137–3148. doi: 10.1093/jxb/erw154. 27122573 PMC4867899

[28] Chen T, Riaz S, Davey P, Zhao Z, Sun Y, Dykes GF, Zhou F, Hartwell J, Lawson T, Nixon PJ. Producing fast and active rubisco in tobacco to enhance photosynthesis. Plant Cell. 2023;35:795–807. doi: 10.1093/plcell/koac348. 36471570 PMC9940876

[29] Schlüter U, Weber APM. Regulation and evolution of C4 photosynthesis. Annu Rev Plant Biol. 2020;71:183–215. doi: 10.1146/annurev-arplant-042916-040915. 32131603

[30] Hultine KR, Cushman JC, Williams DG. New perspectives on crassulacean acid metabolism biology. J Exp Bot. 2019;70:6489–6493. doi: 10.1093/jxb/erz465. 31782509 PMC6883260

[31] Messerschmid TFE, Wehling J, Bobon N, Kahmen A, Klak C, Los JA, Nelson DB, Santos PDos, de Vos JM, Kadereit G. Carbon isotope composition of plant photosynthetic tissues reflects a crassulacean acid metabolism (CAM) continuum in the majority of CAM lineages. Perspect Plant Ecol Evol Syst 2021;51:125619.

[32] Moreno-Villena JJ, Zhou H, Gilman IS, Tausta SL, Cheung CYM, Edwards EJ. Spatial resolution of an integrated C4+ CAM photosynthetic metabolism. Sci Adv. 2022;8:eabn2349. doi: 10.1126/sciadv.abn2349. 35930634 PMC9355352

[33] vanTongerlo E, Trouwborst G, Hogewoning SW, van Ieperen W, Dieleman JA, Marcelis LFM. Crassulacean acid metabolism species differ in the contribution of C3 and C4 carboxylation to end of day CO2 fixation. Physiol Plant. 2021;172:134–145. 33305855 10.1111/ppl.13312 PMC8246577

[34] Winter K, Smith JAC. CAM photosynthesis: the acid test. New Phytol. 2022;233:599–609. doi: 10.1111/nph.17790. 34637529 PMC9298356

[35] Jurić I, Hibberd JM, Blatt M, Burroughs NJ. Computational modelling predicts substantial carbon assimilation gains for C3 plants with a single-celled C4 biochemical pump. PLoS Comput Biol. 2019;15:e1007373. doi: 10.1371/journal.pcbi.1007373. 31568503 PMC6786660

[36] Pradhan B, Panda D, Bishi SK, Chakraborty K, Muthusamy SK, Lenka SK. Progress and prospects of C4 trait engineering in plants. Plant Biol. 2022;24:920–931. doi: 10.1111/plb.13446. 35727191

[37] Karki S, Rizal G, Quick WP. Improvement of photosynthesis in rice (Oryza sativa L.) by inserting the C4 pathway. Rice. 2013;6:1–8. doi: 10.1186/1939-8433-6-28. 24280149 PMC4883725

[38] Ding Z, Weissmann S, Wang M, Du B, Huang L, Wang L, Tu X, Zhong S, Myers C, Brutnell TP. Identification of photosynthesis-associated C4 candidate genes through comparative leaf gradient transcriptome in multiple lineages of C3 and C4 species. PLoS One. 2015;10:e0140629. doi: 10.1371/journal.pone.0140629. 26465154 PMC4605685

[39] Cui H. Challenges and approaches to crop improvement through C3-to-C4 engineering. Front Plant Sci. 2021;12:715391. doi: 10.3389/fpls.2021.715391. 34594351 PMC8476962

[40] Reeves G, Singh P, Rossberg TA, Sogbohossou EOD, Schranz ME, Hibberd JM. Natural variation within a species for traits underpinning C4 photosynthesis. Plant Physiol. 2018;177:504–512. doi: 10.1104/pp.18.00168. 29678862 PMC6001323

[41] Roell M-S, Schada von Borzyskowski L, Westhoff P, Plett A, Paczia N, Claus P, Schlueter U, Erb TJ, Weber APM. A synthetic C4 shuttle via the β-hydroxyaspartate cycle in C3 plants. Proc Natl Acad Sci. 2021;118:e2022307118. doi: 10.1073/pnas.2022307118. 34001608 PMC8166194

[42] Li Y, Heckmann D, Lercher MJ, Maurino VG. Combining genetic and evolutionary engineering to establish C4 metabolism in C3 plants. J Exp Bot. 2017;68:117–125. doi: 10.1093/jxb/erw333. 27660481

[43] Schlüter U, Weber APM. The road to C4 photosynthesis: evolution of a complex trait via intermediary states. Plant Cell Physiol. 2016;57:881–889. doi: 10.1093/pcp/pcw009. 26893471

[44] Jobe TO, Rahimzadeh Karvansara P, Zenzen I, Kopriva S. Ensuring nutritious food under elevated CO2 conditions: a case for improved C4 crops. Front Plant Sci. 2020;11:1267. doi: 10.3389/fpls.2020.01267. 33013946 PMC7461923

[45] Wang L, Du Q, Xie J, Zhou D, Chen B, Yang H, Zhang D. Genetic variation in transcription factors and photosynthesis light-reaction genes regulates photosynthetic traits. Tree Physiol. 2018;38:1871–1885. doi: 10.1093/treephys/tpy079. 30032300

[46] Yu X, Zheng G, Shan L, Meng G, Vingron M, Liu Q, Zhu X-G. Reconstruction of gene regulatory network related to photosynthesis in arabidopsis thaliana. Front Plant Sci. 2014;5:273. doi: 10.3389/fpls.2014.00273. 24982665 PMC4055858

[47] Grabsztunowicz M, Koskela MM, Mulo P. Post-translational modifications in regulation of chloroplast function: recent advances. Front Plant Sci 2017;8:240. doi: 10.3389/fpls.2017.00240. 28280500 PMC5322211

[48] Minagawa J, Tokutsu R. Dynamic regulation of photosynthesis in chlamydomonas reinhardtii. Plant J. 2015;82:413–428. doi: 10.1111/tpj.12805. 25702778

[49] Dehigaspitiya P, Milham P, Ash GJ, Arun-Chinnappa K, Gamage D, Martin A, Nagasaka S, Seneweera S. Exploring natural variation of photosynthesis in a site-specific manner: evolution, progress, and prospects. Planta. 2019;250:1033–1050. doi: 10.1007/s00425-019-03223-1. 31254100

[50] Quero G, Bonnecarrère V, Simondi S, Santos J, Fernández S, Gutierrez L, Garaycochea S, Borsani O. Genetic architecture of photosynthesis energy partitioning as revealed by a genome-wide association approach. Photosynth Res. 2021;150:97–115. doi: 10.1007/s11120-020-00721-2. 32072456

[51] Quan M, Liu X, Du Q, Xiao L, Lu W, Fang Y, Li P, Ji L, Zhang D. Genome-wide association studies reveal the coordinated regulatory networks underlying photosynthesis and wood formation in populus. J Exp Bot. 2021;72:5372–5389. doi: 10.1093/jxb/erab122. 33733665

[52] Li Y, Ren B, Gao L, Ding L, Jiang D, Xu X, Shen Q, Guo S. Less chlorophyll does not necessarily restrain light capture ability and photosynthesis in a chlorophyll‐deficient rice mutant. J Agron Crop Sci. 2013;199:49–56. doi: 10.1111/j.1439-037X.2012.00519.x.

[53] Janeeshma E, Johnson R, Amritha MS, Noble L, Aswathi KPR, Telesiński A, Kalaji HM, Auriga A, Puthur JT. Modulations in chlorophyll a fluorescence based on intensity and spectral variations of light. Int J Mol Sci. 2022;23:5599. doi: 10.3390/ijms23105599. 35628428 PMC9146714

[54] Souza AP, De, Burgess SJ, Doran L, Hansen J, Manukyan L, Maryn N, Gotarkar D, Leonelli L, Niyogi KK, et al. Soybean photosynthesis and crop yield are improved by accelerating recovery from photoprotection. Science (80-). 2022;377:851–854. doi: 10.1126/science.adc9831. 35981033

[55] Cutolo EA, Guardini Z, Dall’Osto L, Bassi R. A paler shade of Green: engineering cellular chlorophyll content to enhance photosynthesis in crowded environments. New Phytol. 2023;239:1567–1583. doi: 10.1111/nph.19064. 37282663

[56] Fiebig OC, Harris D, Wang D, Hoffmann MP, Schlau-Cohen GS. Ultrafast dynamics of photosynthetic light harvesting: strategies for acclimation across organisms. Annu Rev Phys Chem. 2023;74:493–520. doi: 10.1146/annurev-physchem-083122-111318. 36791782

[57] Negi S, Perrine Z, Friedland N, Kumar A, Tokutsu R, Minagawa J, Berg H, Barry AN, Govindjee G, Sayre R. Light regulation of light‐harvesting antenna size substantially enhances photosynthetic efficiency and biomass yield in Green algae. Plant J. 2020;103:584–603. doi: 10.1111/tpj.14751. 32180283

[58] Roach T, Kim E, Tian L, Lepetit B. Editorial: regulation of light-harvesting systems during acclimation of photosynthetic organisms. Front Plant Sci. 2022;13:914047. doi: 10.3389/fpls.2022.914047. 35599876 PMC9118192

[59] Zhen S, Bugbee B. Substituting far-red for traditionally defined photosynthetic photons results in equal canopy quantum yield for CO2 fixation and increased photon capture during long-term studies: implications for re-defining PAR. Front Plant Sci. 2020;11:581156. doi: 10.3389/fpls.2020.581156. 33014004 PMC7516038

[60] Gollan PJ, Lima-Melo Y, Tiwari A, Tikkanen M, Aro E-M. Interaction between photosynthetic electron transport and chloroplast sinks triggers protection and signalling important for plant productivity. Philos Trans R Soc B Biol Sci. 2017;372:20160390. doi: 10.1098/rstb.2016.0390. PMC5566885 28808104

[61] Ma M, Liu Y, Bai C, Yang Y, Sun Z, Liu X, Zhang S, Han X, Yong JWH. The physiological functionality of PGR5/PGRL1-dependent cyclic electron transport in sustaining photosynthesis. Front Plant Sci. 2021;12:702196. doi: 10.3389/fpls.2021.702196. 34305990 PMC8294387

[62] Yamori W, Shikanai T. Physiological functions of cyclic electron transport around photosystem I in sustaining photosynthesis and plant growth. Annu Rev Plant Biol. 2016;67:81–106. doi: 10.1146/annurev-arplant-043015-112002. 26927905

[63] Storti M, Alboresi A, Gerotto C, Aro E, Finazzi G, Morosinotto T. Role of cyclic and pseudo‐cyclic electron transport in response to dynamic light changes in physcomitrella patens. Plant Cell Environ. 2019;42:1590–1602. doi: 10.1111/pce.13493. 30496624

[64] Taylor CR, van Ieperen W, Harbinson J. Demonstration of a relationship between state transitions and photosynthetic efficiency in a higher plant. Biochem J 2019;476:3295–3312. 31694051 10.1042/BCJ20190576 PMC6854431

[65] Kramer M, Rodriguez-Heredia M, Saccon F, Mosebach L, Twachtmann M, Krieger-Liszkay A, Duffy C, Knell RJ, Finazzi G, Hanke GT. Regulation of photosynthetic electron flow on dark to light transition by ferredoxin: NADP (H) oxidoreductase interactions. eLife. 2021;10:e56088. doi: 10.7554/eLife.56088. 33685582 PMC7984839

[66] Morales A, Yin X, Harbinson J, Driever SM, Molenaar J, Kramer DM, Struik PC. In silico analysis of the regulation of the photosynthetic electron transport chain in C3 plants. Plant Physiol. 2018;176:1247–1261. doi: 10.1104/pp.17.00779. 28924017 PMC5813522

[67] Fernie AR, Bauwe H. Wasteful, essential, evolutionary stepping stone? The multiple personalities of the photorespiratory pathway. Plant J. 2020;102:666–677. 31904886 10.1111/tpj.14669

[68] Timm S, Hagemann M. Photorespiration—how is it regulated and how does it regulate overall plant metabolism? J Exp Bot. 2020;71:3955–3965. doi: 10.1093/jxb/eraa183. 32274517

[69] Timm S, Florian A, Fernie AR, Bauwe H. The regulatory interplay between photorespiration and photosynthesis. J Exp Bot. 2016;67:2923–2929. doi: 10.1093/jxb/erw083. 26969745

[70] Reinholdt O, Schwab S, Zhang Y, Reichheld J-P, Fernie AR, Hagemann M, Timm S. Redox-regulation of photorespiration through mitochondrial thioredoxin o1. Plant Physiol. 2019;181:442–457. doi: 10.1104/pp.19.00559. 31413204 PMC6776843

[71] Busch FA. Photorespiration in the context of rubisco biochemistry, CO2 diffusion and metabolism. Plant J. 2020;101:919–939. 31910295 10.1111/tpj.14674

[72] Florian A, Araújo WL, Fernie AR. New insights into photorespiration obtained from metabolomics. Plant Biol. 2013;15:656–666. doi: 10.1111/j.1438-8677.2012.00704.x. 23573870

[73] Cummins PL. The coevolution of RuBisCO, photorespiration, and carbon concentrating mechanisms in higher plants. Front Plant Sci. 2021;12:662425. 34539685 10.3389/fpls.2021.662425 PMC8440988

[74] Orr DJ, Robijns AKJ, Baker CR, Niyogi KK, Carmo-Silva E. Dynamics of rubisco regulation by sugar phosphate derivatives and their phosphatases. J Exp Bot. 2023;74:581–590. doi: 10.1093/jxb/erac386. 36173669 PMC9833046

[75] Tcherkez G. The mechanism of rubisco‐catalysed oxygenation. Plant Cell Environ. 2016;39:983–997. doi: 10.1111/pce.12629. 26286702

[76] Bloom AJ, Lancaster KM. Manganese binding to rubisco could drive a photorespiratory pathway that increases the energy efficiency of photosynthesis. Nat Plants. 2018;4:414–422. doi: 10.1038/s41477-018-0191-0. 29967515

[77] Davis GA, Kramer DM. Optimization of ATP synthase c–rings for oxygenic photosynthesis. Front Plant Sci. 2020;10:1778. doi: 10.3389/fpls.2019.01778. 32082344 PMC7003800

[78] Yamamoto H, Cheuk A, Shearman J, Nixon PJ, Meier T, Shikanai T. Impact of engineering the ATP synthase rotor ring on photosynthesis in tobacco chloroplasts. Plant Physiol. 2023;192:1221–1233. doi: 10.1093/plphys/kiad043. 36703219 PMC10231360

[79] Ermakova M, Heyno E, Woodford R, Massey B, Birke H, Caemmerer S, Von. Enhanced abundance and activity of the chloroplast ATP synthase in rice through the overexpression of the AtpD subunit. J Exp Bot. 2022;73:6891–6901. doi: 10.1093/jxb/erac320. 35904136 PMC9629782

[80] Lou W, Tan X, Song K, Zhang S, Luan G, Li C, Lu X. A specific single nucleotide polymorphism in the ATP synthase gene significantly improves environmental stress tolerance of synechococcus elongatus PCC 7942. Appl Environ Microbiol. 2018;84(18):e01222. doi: 10.1128/AEM.01222-18. 30006407 PMC6121992

[81] Jin K, Chen G, Yang Y, Zhang Z, Lu T. Strategies for manipulating rubisco and creating photorespiratory bypass to boost C3 photosynthesis: prospects on modern crop improvement. Plant Cell Environ. 2023;46:363–378. doi: 10.1111/pce.14500. 36444099

[82] Li R, He Y, Chen J, Zheng S, Zhuang C. Research progress in improving photosynthetic efficiency. Int J Mol Sci. 2023;24:9286. doi: 10.3390/ijms24119286. 37298238 PMC10253142

[83] Schöttler MA, Tóth SZ, Boulouis A, Kahlau S. Photosynthetic complex stoichiometry dynamics in higher plants: biogenesis, function, and turnover of ATP synthase and the cytochrome b 6 f complex. J Exp Bot. 2015;66:2373–2400. 25540437 10.1093/jxb/eru495

[84] Zhang Y, Tian L, Lu C. Chloroplast gene expression: recent advances and perspectives. Plant Commun. 2023;4:100611. doi: 10.1016/j.xplc.2023.100611. 37147800 PMC10504595

[85] Berry JO, Yerramsetty P, Zielinski AM, Mure CM. Photosynthetic gene expression in higher plants. Photosynth Res. 2013;117:91–120. doi: 10.1007/s11120-013-9880-8. 23839301

[86] Oh S, Montgomery BL. Phytochrome-dependent coordinate control of distinct aspects of nuclear and plastid gene expression during anterograde signaling and photomorphogenesis. Front Plant Sci. 2014;5:171. doi: 10.3389/fpls.2014.00171. 24817873 PMC4012200

[87] Sun Y, Yao Z, Ye Y, Fang J, Chen H, Lyu Y, Broad W, Fournier M, Chen G, Hu Y. Ubiquitin-based pathway acts inside chloroplasts to regulate photosynthesis. Sci Adv. 2022;8:eabq7352. doi: 10.1126/sciadv.abq7352. 36383657 PMC9668298

[88] Yang EJ, Yoo CY, Liu J, Wang H, Cao J, Li F-W, Pryer KM, Sun T, Weigel D, Zhou P. NCP activates chloroplast transcription by controlling phytochrome-dependent dual nuclear and plastidial switches. Nat Commun. 2019;10:2630. doi: 10.1038/s41467-019-10517-1. 31201314 PMC6570768

[89] Jin H, Fu M, Duan Z, Duan S, Li M, Dong X, Liu B, Feng D, Wang J, Peng L. Low photosynthetic efficiency 1 is required for light-regulated photosystem II biogenesis in arabidopsis. Proc Natl Acad Sci. 2018;115:E6075–E6084. doi: 10.1073/pnas.1807364115. 29891689 PMC6042084

[90] Shrestha B, Gilbert LE, Ruhlman TA, Jansen RK. Rampant nuclear transfer and substitutions of plastid genes in passiflora. Genome Biol Evol. 2020;12:1313–1329. doi: 10.1093/gbe/evaa123. 32539116 PMC7488351

[91] Jing Y, Lin R. Transcriptional regulatory network of the light signaling pathways. New Phytol. 2020;227:683–697. doi: 10.1111/nph.16602. 32289880

[92] Toledo-Ortiz G, Johansson H, Lee KP, Bou-Torrent J, Stewart K, Steel G, Rodríguez-Concepción M, Halliday KJ. The HY5-PIF regulatory module coordinates light and temperature control of photosynthetic gene transcription. PLoS Genet. 2014a;10:e1004416. doi: 10.1371/journal.pgen.1004416. 24922306 PMC4055456

[93] Hernández-Prieto MA, Semeniuk TA, Giner-Lamia J, Futschik ME. The transcriptional landscape of the photosynthetic model cyanobacterium synechocystis sp. Pcc6803. Sci Rep. 2016;6:22168. doi: 10.1038/srep22168. 26923200 PMC4770689

[94] Taylor EB. Metabolic control of transcription. Science (80-). 2023;381:125–126. doi: 10.1126/science.adi7577. 37440640

[95] Wang P, Hendron R, Kelly S. Transcriptional control of photosynthetic capacity: conservation and divergence from arabidopsis to rice. New Phytol. 2017;216:32–45. doi: 10.1111/nph.14682. 28727145

[96] Imam S, Noguera DR, Donohue TJ. Global analysis of photosynthesis transcriptional regulatory networks. PLoS Genet. 2014;10:e1004837. doi: 10.1371/journal.pgen.1004837. 25503406 PMC4263372

[97] Slobodin B, Dikstein R. So close, no matter how far: multiple paths connecting transcription to mRNA translation in eukaryotes. EMBO Rep. 2020;21:e50799. 32803873 10.15252/embr.202050799 PMC7507372

[98] Gururani MA. Photobiotechnology for abiotic stress resilient crops: recent advances and prospects. Heliyon. 2023;9:e20158. doi: 10.1016/j.heliyon.2023.e20158. 37810087 PMC10559926

[99] Toledo-Ortiz G, Johansson H, Lee KP, Bou-Torrent J, Stewart K, Steel G, Rodríguez-Concepción M, Halliday KJ. The HY5-PIF regulatory module coordinates light and temperature control of photosynthetic gene transcription. PLoS Genet. 2014b;10:e1004416. doi: 10.1371/journal.pgen.1004416. 24922306 PMC4055456

[100] Kaiser E, Morales A, Harbinson J, Kromdijk J, Heuvelink E, Marcelis LFM. Dynamic photosynthesis in different environmental conditions. J Exp Bot. 2015;66:2415–2426. doi: 10.1093/jxb/eru406. 25324402

[101] Demmig-Adams B, Stewart JJ, Baker CR, Adams III WW. Optimization of photosynthetic productivity in contrasting environments by regulons controlling plant form and function. Int J Mol Sci. 2018;19:872. doi: 10.3390/ijms19030872. 29543762 PMC5877733

[102] Borbély P, Gasperl A, Pálmai T, Ahres M, Asghar MA, Galiba G, Müller M, Kocsy G. Light intensity-and spectrum-dependent redox regulation of plant metabolism. Antioxidants. 2022;11:1311. doi: 10.3390/antiox11071311. 35883801 PMC9312225

[103] Alameldin HF, Montgomery BL. Plasticity of arabidopsis rosette transcriptomes and photosynthetic responses in dynamic light conditions. Plant Direct. 2023;7:e475. doi: 10.1002/pld3.475. 36628154 PMC9822700

[104] Ali AA, Xu C, Rogers A, McDowell NG, Medlyn BE, Fisher RA, Wullschleger SD, Reich PB, Vrugt JA, Bauerle WL. Global‐scale environmental control of plant photosynthetic capacity. Ecol Appl. 2015;25:2349–2365. doi: 10.1890/14-2111.1. 26910960

[105] Keller B, Matsubara S, Rascher U, Pieruschka R, Steier A, Kraska T, Muller O. Genotype specific photosynthesis x environment interactions captured by automated fluorescence canopy scans over two fluctuating growing seasons. Front Plant Sci. 2019;10:1482. doi: 10.3389/fpls.2019.01482. 31998328 PMC6962999

[106] Longoni FP, Goldschmidt-Clermont M. Thylakoid protein phosphorylation in chloroplasts. Plant Cell Physiol. 2021;62:1094–1107. doi: 10.1093/pcp/pcab043. 33768241

[107] Longoni P, Douchi D, Cariti F, Fucile G, Goldschmidt-Clermont M. Phosphorylation of the light-harvesting complex II isoform Lhcb2 is central to state transitions. Plant Physiol. 2015;169(4):2874–2883. doi: 10.1104/pp.15.01498. 26438789 PMC4677923

[108] Kirst H, Gabilly ST, Niyogi KK, Lemaux PG, Melis A. Photosynthetic antenna engineering to improve crop yields. Planta. 2017;245:1009–1020. doi: 10.1007/s00425-017-2659-y. 28188423

[109] Li W, Wu S, Zhang H, Zhang X, Zhuang J, Hu C, Liu Y, Lei B, Ma L, Wang X. Enhanced biological photosynthetic efficiency using light‐harvesting engineering with dual‐emissive carbon dots. Adv Funct Mater. 2018;28:1804004. doi: 10.1002/adfm.201804004.

[110] Xu Y, Fei J, Li G, Yuan T, Xu X, Wang C, Li J. Optically matched semiconductor quantum dots improve photophosphorylation performed by chloroplasts. Angew Chemie Int Ed. 2018;57:6532–6535. doi: 10.1002/anie.201802555. 29655302

[111] Betterle N, Ballottari M, Baginsky S, Bassi R. High light-dependent phosphorylation of photosystem II inner antenna CP29 in monocots is STN7 independent and enhances nonphotochemical quenching. Plant Physiol. 2015;167:457–471. doi: 10.1104/pp.114.252379. 25501945 PMC4326754

[112] Koskela MM, Brünje A, Ivanauskaite A, Grabsztunowicz M, Lassowskat I, Neumann U, Dinh TV, Sindlinger J, Schwarzer D, Wirtz M. Chloroplast acetyltransferase NSI is required for state transitions in arabidopsis thaliana. Plant Cell. 2018;30:1695–1709. doi: 10.1105/tpc.18.00155. 29967049 PMC6139681

[113] Rantala M, Ivanauskaite A, Laihonen L, Kanna SD, Ughy B, Mulo P. Chloroplast acetyltransferase GNAT2 is involved in the organization and dynamics of thylakoid structure. Plant Cell Physiol. 2022;63:1205–1214. doi: 10.1093/pcp/pcac096. 35792507 PMC9474947

[114] Ivanauskaite A, Rantala M, Laihonen L, Konert MM, Schwenner N, Mühlenbeck JS, Finkemeier I, Mulo P. Loss of chloroplast GNAT acetyltransferases results in distinct metabolic phenotypes in arabidopsis. Plant Cell Physiol. 2023;64:549–563. doi: 10.1093/pcp/pcad017. 37026998 PMC10190055

[115] Bai T, Guo L, Xu M, Tian L. Structural diversity of photosystem I and its light-harvesting system in eukaryotic algae and plants. Front Plant Sci. 2021;12:781035. doi: 10.3389/fpls.2021.781035. 34917114 PMC8669154

[116] Jr, Balevičius V, Fox KF, Bricker WP, Jurinovich S, Prandi IG, Mennucci B, Duffy CDP. Fine control of chlorophyll-carotenoid interactions defines the functionality of light-harvesting proteins in plants. Sci Rep. 2017;7:13956. doi: 10.1038/s41598-017-13720-6. 29066753 PMC5655323

[117] Dietz K-J, Hell R. Thiol switches in redox regulation of chloroplasts: balancing redox state, metabolism and oxidative stress. Biol Chem. 2015;396:483–494. 25741945 10.1515/hsz-2014-0281

[118] Dinakar C, Vishwakarma A, Raghavendra AS, Padmasree K. Alternative oxidase pathway optimizes photosynthesis during osmotic and temperature stress by regulating cellular ROS, malate valve and antioxidative systems. Front Plant Sci. 2016;7:68. 26904045 10.3389/fpls.2016.00068 PMC4747084

[119] Karlusich JJP, Arce RC, Shahinnia F, Sonnewald S, Sonnewald U, Zurbriggen MD, Hajirezaei M-R, Carrillo N. Transcriptional and metabolic profiling of potato plants expressing a plastid-targeted electron shuttle reveal modulation of genes associated to drought tolerance by chloroplast redox poise. Int J Mol Sci. 2020;21:7199. doi: 10.3390/ijms21197199. 33003500 PMC7582712

[120] Hancock AM, Son M, Nairat M, Wei T, Jeuken LJC, Duffy CDP, Schlau-Cohen GS, Adams PG. Ultrafast energy transfer between lipid-linked chromophores and plant light-harvesting complex II. Phys Chem Chem Phys. 2021;23:19511–19524. doi: 10.1039/D1CP01628H. 34524278 PMC8442836

[121] Pan X, Ma J, Su X, Cao P, Chang W, Liu Z, Zhang X, Li M. Structure of the maize photosystem I supercomplex with light-harvesting complexes I and II. Science (80-.). 2018;360:1109–1113. doi: 10.1126/science.aat1156. 29880686

[122] Xu D-Q, Chen Y, Chen G-Y. Light-harvesting regulation from leaf to molecule with the emphasis on rapid changes in antenna size. Photosynth Res. 2015;124:137–158. doi: 10.1007/s11120-015-0115-z. 25773873

[123] Allen JF. Why we need to know the structure of phosphorylated chloroplast light‐harvesting complex II. Physiol Plant. 2017;161:28–44. doi: 10.1111/ppl.12577. 28393369

[124] Liguori N, Periole X, Marrink SJ, Croce R. From light-harvesting to photoprotection: structural basis of the dynamic switch of the major antenna complex of plants (LHCII). Sci Rep. 2015;5:15661. doi: 10.1038/srep15661. 26493782 PMC4616226

[125] Liguori N, Xu P, Van Stokkum IHM, Van Oort B, Lu Y, Karcher D, Bock R, Croce R. Different carotenoid conformations have distinct functions in light-harvesting regulation in plants. Nat Commun. 2017;8 1994. 29222488 10.1038/s41467-017-02239-z PMC5722816

[126] Mascoli V, Liguori N, Cupellini L, Elias E, Mennucci B, Croce R. Uncovering the interactions driving carotenoid binding in light-harvesting complexes. Chem Sci. 2021;12:5113–5122. doi: 10.1039/D1SC00071C. 34163750 PMC8179543

[127] Riaz A, Deng F, Chen G, Jiang W, Zheng Q, Riaz B, Mak M, Zeng F, Chen Z-H. Molecular regulation and evolution of redox homeostasis in photosynthetic machinery. Antioxidants. 2022;11:2085. doi: 10.3390/antiox11112085. 36358456 PMC9686623

[128] Cardol P, Krieger‐Liszkay A. From light capture to metabolic needs, oxygenic photosynthesis is an ever‐expanding field of study in plants, algae and Cyanobacteria. Physiol Plant. 2017;161:2–5. doi: 10.1111/ppl.12589. 28547911

[129] Wu A, Brider J, Busch FA, Chen M, Chenu K, Clarke VC, Collins B, Ermakova M, Evans JR, Farquhar GD. A cross‐scale analysis to understand and quantify the effects of photosynthetic enhancement on crop growth and yield across environments. Plant Cell Environ. 2023;46:23–44. doi: 10.1111/pce.14453. 36200623 PMC10091820

[130] Johnson SL. A year at the forefront of engineering photosynthesis. Biol Open. 2022;11:bio059335. doi: 10.1242/bio.059335. 35876381 PMC9346289

[131] Kromdijk J, Long SP. One crop breeding cycle from starvation? How engineering crop photosynthesis for rising CO2 and temperature could be one important route to alleviation. Proc R Soc B Biol Sci. 2016;283:20152578. doi: 10.1098/rspb.2015.2578. PMC4810849 26962136

[132] Liu D, Hu R, Zhang J, Guo H-B, Cheng H, Li L, Borland AM, Qin H, Chen J-G, Muchero W. Overexpression of an agave phospho enol pyruvate carboxylase improves plant growth and stress tolerance. Cells. 2021;10:582. doi: 10.3390/cells10030582. 33800849 PMC7999111

[133] Tahjib-Ul-Arif M, Zahan MI, Karim MM, Imran S, Hunter CT, Islam MS, Mia MA, Hannan MA, Rhaman MS, Hossain MA. Citric acid-mediated abiotic stress tolerance in plants. Int J Mol Sci. 2021;22:7235. doi: 10.3390/ijms22137235. 34281289 PMC8268203

[134] Nishiyama Y, Murata N. Revised scheme for the mechanism of photoinhibition and its application to enhance the abiotic stress tolerance of the photosynthetic machinery. Appl Microbiol Biotechnol. 2014;1:8777–8796. doi: 10.1007/s00253-014-6020-0. 25139449

[135] Amin AB, Rathnayake KN, Yim WC, Garcia TM, Wone B, Cushman JC, Wone BWM. Crassulacean acid metabolism abiotic stress-responsive transcription factors: a potential genetic engineering approach for improving crop tolerance to abiotic stress. Front Plant Sci. 2019;10:129. doi: 10.3389/fpls.2019.00129. 30853963 PMC6395430

[136] Gururani MA, Mohanta TK, Bae H. Current understanding of the interplay between phytohormones and photosynthesis under environmental stress. Int J Mol Sci. 2015a;16:19055–19085. doi: 10.3390/ijms160819055. 26287167 PMC4581286

[137] Zhao LS, Li K, Wang QM, Song XY, Su HN, Xie BBin, Zhang XY, Huang F, Chen XL, Zhou BC, et al. Nitrogen starvation impacts the photosynthetic performance of porphyridium cruentum as revealed by chlorophyll a fluorescence. Sci Rep. 2017;7:1–11. doi: 10.1038/s41598-017-08428-6. 28819147 PMC5561210

[138] Broad RC, Bonneau JP, Hellens RP, Johnson AAT. Manipulation of ascorbate biosynthetic, recycling, and regulatory pathways for improved abiotic stress tolerance in plants. Int J Mol Sci. 2020;21:1790. 32150968 10.3390/ijms21051790 PMC7084844

[139] Yu L, Chen Q, Peng Y, Xie L, Liu D, Han M, Chen F, Xiao S, Huang J, Li J. Arabidopsis thaliana plants engineered to produce astaxanthin show enhanced oxidative stress tolerance and bacterial pathogen resistance. J Agric Food Chem. 2019;67:12590–12598. doi: 10.1021/acs.jafc.9b04589. 31639305

[140] Jansson C, Vogel J, Hazen S, Brutnell T, Mockler T. Climate-smart crops with enhanced photosynthesis. J Exp Bot. 2018;69:3801–3809. doi: 10.1093/jxb/ery213. 30032188

[141] Wu A, Hammer GL, Doherty A, von Caemmerer S, Farquhar GD. Quantifying impacts of enhancing photosynthesis on crop yield. Nat Plants. 2019;5:380–388. 30962528 10.1038/s41477-019-0398-8

[142] Krewald V, Retegan M, Pantazis DA. Principles of natural photosynthesis. Sol. Energy Fuels. 2016;23:48. 10.1007/128_2015_645 26099285

[143] Zhao S, Zhang Q, Liu M, Zhou H, Ma C, Wang P. Regulation of plant responses to salt stress. Int J Mol Sci. 2021;22:1–16. doi: 10.3390/ijms22094609. PMC8125386 33924753

[144] Mora SJ, Odella E, Moore GF, Gust D, Moore TA, Moore AL. Proton-coupled electron transfer in artificial photosynthetic systems. Acc Chem Res. 2018;51:445–453. doi: 10.1021/acs.accounts.7b00491. 29309118

[145] Kim Y, Lee JH, Ha H, Im SW, Nam KT. Material science lesson from the biological photosystem. Nano Converg. 2016;3:1–11. doi: 10.1186/s40580-016-0079-5. 28191429 PMC5271162

[146] Yoneda Y, Noji T, Mizutani N, Kato D, Kondo M, Miyasaka H, Nagasawa Y, Dewa T. Energy transfer dynamics and the mechanism of biohybrid photosynthetic antenna complexes chemically linked with artificial chromophores. Phys Chem Chem Phys. 2022;24:24714–24726. doi: 10.1039/D2CP02465A. 36128743

[147] Kathpalia R, Verma AK. Artificial photosynthesis as an alternative source of renewable energy: potential and limitations. In Plant Physiology Annual Volume 2023. 2023. UNITED KINGDOM: IntechOpen.

[148] Ren Y-Y, Wang F. Supramolecular artificial photosynthetic systems: from assembly to bionics. Curr. Opin. Green Sustain. Chem. 2023;41:100808. doi: 10.1016/j.cogsc.2023.100808.

[149] Ort DR, Merchant SS, Alric J, Barkan A, Blankenship RE, Bock R, Croce R, Hanson MR, Hibberd JM, Long SP. Redesigning photosynthesis to sustainably meet global food and bioenergy demand. Proc Natl Acad Sci. 2015;112:8529–8536. doi: 10.1073/pnas.1424031112. 26124102 PMC4507207

[150] Tomo T, Allakhverdiev SI. Preface: photosynthesis and hydrogen energy research for sustainability. Photosynth Res. 2017;133(1–3):1–3. doi: 10.1007/s11120-017-0378-7. 28396976

[151] Janssen PJD, Lambreva MD, Plumeré N, Bartolucci C, Antonacci A, Buonasera K, Frese RN, Scognamiglio V, Rea G. Photosynthesis at the forefront of a sustainable life. Front Chem. 2014;2:36. doi: 10.3389/fchem.2014.00036. 24971306 PMC4054791

[152] Niinemets Ü, Berry JA, Caemmerer S, von, Ort DR, Parry MAJ, Poorter H. Photosynthesis: ancient, essential, complex, diverse… and in need of improvement in a changing world. New Phytol. 2017;213:43–47. doi: 10.1111/nph.14307. 27891642

[153] Cruz JA, Savage LJ, Zegarac R, Hall CC, Satoh-Cruz M, Davis GA, Kovac WK, Chen J, Kramer DM. Dynamic environmental photosynthetic imaging reveals emergent phenotypes. Cell Syst. 2016;2:365–377. doi: 10.1016/j.cels.2016.06.001. 27336966

[154] McAusland L, Atkinson JA, Lawson T, Murchie EH. High throughput procedure utilising chlorophyll fluorescence imaging to phenotype dynamic photosynthesis and photoprotection in leaves under controlled gaseous conditions. Plant Methods. 2019;15:1–15. doi: 10.1186/s13007-019-0485-x. 31548849 PMC6749646

[155] Bezouw RFHM, van, Keurentjes JJB, Harbinson J, Aarts MGM. Converging phenomics and genomics to study natural variation in plant photosynthetic efficiency. Plant J. 2019;97:112–133. doi: 10.1111/tpj.14190. 30548574 PMC6850172

[156] Flood PJ, Kruijer W, Schnabel SK, Schoor R, van der, Jalink H, Snel JFH, Harbinson J, Aarts MGM. Phenomics for photosynthesis, growth and reflectance in arabidopsis thaliana reveals circadian and long-term fluctuations in heritability. Plant Methods. 2016;12(1):14. doi: 10.1186/s13007-016-0113-y. 26884806 PMC4754911

[157] Fu P, Meacham-Hensold K, Siebers MH, Bernacchi CJ. The inverse relationship between solar-induced fluorescence yield and photosynthetic capacity: benefits for field phenotyping. J Exp Bot. 2021;72:1295–1306. doi: 10.1093/jxb/eraa537. 33340310 PMC7904154

[158] Song Q, Chu C, Parry MAJ, Zhu X. Genetics‐based dynamic systems model of canopy photosynthesis: the key to improve light and resource use efficiencies for crops. Food Energy Secur. 2016;5:18–25. doi: 10.1002/fes3.74. 27867502 PMC5108349

[159] Abebe AM, Kim Y, Kim J, Kim SL, Baek J. Image-based high-throughput phenotyping in horticultural crops. Plants. 2023;12:2061. doi: 10.3390/plants12102061. 37653978 PMC10222289

[160] Sukhova E, Ratnitsyna D, Gromova E, Sukhov V. Development of two-dimensional model of photosynthesis in plant leaves and analysis of induction of spatial heterogeneity of CO2 assimilation rate under action of excess light and drought. Plants. 2022;11:3285. doi: 10.3390/plants11233285. 36501325 PMC9739240

[161] Sundermann EM, Lercher MJ, Heckmann D. Modeling photosynthetic resource allocation connects physiology with evolutionary environments. Sci Rep. 2021;11:15979. doi: 10.1038/s41598-021-94903-0. 34354112 PMC8342476

[162] Ding H, Wang Z, Zhang Y, Li J, Jia L, Chen Q, Ding Y, Wang S. A mechanistic model for estimating rice photosynthetic capacity and stomatal conductance from sun-induced chlorophyll fluorescence. Plant Phenomics. 2023;5:47. doi: 10.34133/plantphenomics.0047. PMC10204737 37228514

[163] Gu L, Han J, Wood JD, Chang CY, Sun Y. Sun‐induced chl fluorescence and its importance for biophysical modeling of photosynthesis based on light reactions. New Phytol. 2019;223:1179–1191. doi: 10.1111/nph.15796. 30883811

[164] Bernacchi CJ, Bagley JE, Serbin SP, Ruiz‐Vera UM, Rosenthal DM, Vanloocke A. Modelling C 3 photosynthesis from the chloroplast to the ecosystem. Plant Cell Environ. 2013;36:1641–1657. doi: 10.1111/pce.12118. 23590343

[165] Ubierna N, Cernusak LA. Preface: advances in modelling photosynthetic processes in terrestrial plants. Photosynth Res. 2019;141:1–3. doi: 10.1007/s11120-019-00651-8. 31209643

[166] Mahmood U, Li X, Fan Y, Chang W, Niu Y, Li J, Qu C, Lu K. Multi-omics revolution to promote plant breeding efficiency. Front Plant Sci. 2022;13:1062952. doi: 10.3389/fpls.2022.1062952. 36570904 PMC9773847

[167] Yang Y, Saand MA, Huang L, Abdelaal WB, Zhang J, Wu Y, Li J, Sirohi MH, Wang F. Applications of multi-omics technologies for crop improvement. Front Plant Sci. 2021;12:563953. doi: 10.3389/fpls.2021.563953. 34539683 PMC8446515

[168] Pazhamala LT, Kudapa H, Weckwerth W, Millar AH, Varshney RK. Systems biology for crop improvement. Plant Genome. 2021;14:e20098. doi: 10.1002/tpg2.20098. 33949787 PMC12806876

[169] Mathan J, Bhattacharya J, Ranjan A. Enhancing crop yield by optimizing plant developmental features. Development. 2016;143:3283–3294. doi: 10.1242/dev.134072. 27624833

[170] Baslam M, Mitsui T, Hodges M, Priesack E, Herritt MT, Aranjuelo I, Sanz-Sáez Á. Photosynthesis in a changing global climate: scaling up and scaling down in crops. Front Plant Sci. 2020;11:882. doi: 10.3389/fpls.2020.00882. 32733499 PMC7357547

[171] Dikobe T, Masenya K, Manganyi MC. Molecular technologies ending with ‘omics’: the driving force toward sustainable plant production and protection. F1000Research. 2023;12:480. doi: 10.12688/f1000research.131413.1.

[172] Amin A, Zaman W, Park S. Harnessing multi-omics and predictive modeling for climate-resilient crop breeding: from genomes to fields. Genes. 2025;16(7):809. doi: 10.3390/genes16070809. 40725465 PMC12294880

[173] Duan F, Li X, Wei Z, Li J, Jiang C, Jiao C, Zhao S, Kong Y, Yan M, Huang J, et al. Multi-omics analysis reveals distinct responses to light stress in photosynthesis and primary metabolism between maize and rice. Plant Commun. 2025;6(10):101488. doi: 10.1016/j.xplc.2025.101488. 40836430 PMC12546642

[174] Zhu T, Li T, Lü P, Li C. Single-cell omics in plant biology: mechanistic insights and applications for crop improvement. Adv Biotechnol. 2025;3:20. doi: 10.1007/s44307-025-00074-8. PMC12214168 40593253

[175] Bradbury A, Clapp O, Biacsi AS, Kuo P, Gaju O, Hayta S, Zhu JK, Lambing C. Integrating genome editing with omics, artificial intelligence, and advanced farming technologies to increase crop productivity. Plant Commun. 2025;14(7):101386 10.1016/j.xplc.2025.101386 PMC12281252 40443034

[176] Chen F, Chen L, Yan Z, Xu J, Feng L, He N, Guo M, Zhao J, Chen Z, Chen H, et al. Recent advances of CRISPR-based genome editing for enhancing staple crops. Front Plant Sci. 2024;15:1478398. doi: 10.3389/fpls.2024.1478398. 39376239 PMC11456538

[177] Tripodi P. Next generation breeding in the omics era. Reprod Breed. 2025;5(2):88–91. doi: 10.1016/j.repbre.2025.05.001.

[178] Zhang Y, Huang G, Zhao Y, Lu X, Wang Y, Wang C, Guo X, Zhao C. Revolutionizing crop breeding: next-generation artificial intelligence and big data-driven intelligent design. Engineering. 2025;44:245–255. doi: 10.1016/j.eng.2024.11.034.

[179] Araus JL, Sanchez-Bragado R, Vicente R. Improving crop yield and resilience through optimization of photosynthesis: panacea or pipe dream? J Exp Bot. 2021;72:3936–3955. doi: 10.1093/jxb/erab097. 33640973

[180] Mushtaq MA, Ateeq M, Ikram M, Alam SM, Kaleem MM, Ashraf MA, Asim M, Almutairi KF, Seleiman MF, Shireen F. Securing fruit trees future: AI-driven early warning and predictive systems for abiotic stress in changing climate. Plant Stress. 2025;17:100953. doi: 10.1016/j.stress.2025.100953.

[181] Theeuwen TPJM, Logie LL, Harbinson J, Aarts MGM. Genetics as a key to improving crop photosynthesis. J Exp Bot. 2022;73:3122–3137. doi: 10.1093/jxb/erac076. 35235648 PMC9126732

[182] Furbank RT, Sharwood R, Estavillo GM, Silva-Perez V, Condon AG. Photons to food: genetic improvement of cereal crop photosynthesis. J Exp Bot. 2020;71:2226–2238. doi: 10.1093/jxb/eraa077. 32083680 PMC7135014

[183] Iñiguez C, Aguiló-Nicolau P, Galmés J. Improving photosynthesis through the enhancement of rubisco carboxylation capacity. Biochem Soc Trans. 2021;49:2007–2019. doi: 10.1042/BST20201056. 34623388

[184] Price GD, Howitt SM. Towards turbocharged photosynthesis. Natur. 2014;513:497–498. doi: 10.1038/nature13749. 25231859

[185] Price GD, Pengelly JJL, Forster B, Du J, Whitney SM, Caemmerer S, Von, Badger MR, Howitt SM, Evans JR. The cyanobacterial CCM as a source of genes for improving photosynthetic CO2 fixation in crop species. J Exp Bot. 2013;64:753–768. doi: 10.1093/jxb/ers257. 23028015

[186] Dann M, Leister D. Enhancing (crop) plant photosynthesis by introducing novel genetic diversity. Philos. Trans. R. Soc. B Biol. Sci. 2017;372:20160380. doi: 10.1098/rstb.2016.0380. PMC5566880 28808099

[187] Zheng S, Ye C, Lu J, Liufu J, Lin L, Dong Z, Li J, Zhuang C. Improving the rice photosynthetic efficiency and yield by editing OsHXK1 via CRISPR/Cas9 system. Int J Mol Sci. 2021;22:9554. doi: 10.3390/ijms22179554. 34502462 PMC8430575

[188] Tiwari JK, Singh AK, Behera TK. CRISPR/Cas genome editing in tomato improvement: advances and applications. Front Plant Sci. 2023;14:1121209. doi: 10.3389/fpls.2023.1121209. 36909403 PMC9995852

[189] Hussain B, Lucas SJ, Budak H. CRISPR/Cas9 in plants: at play in the genome and at work for crop improvement. Brief Funct Genomics. 2018;17:319–328. doi: 10.1093/bfgp/ely016. 29912293

[190] Rao MJ, Wang L. CRISPR/Cas9 technology for improving agronomic traits and future prospective in agriculture. Planta. 2021;254:1–16. doi: 10.1007/s00425-021-03716-y. 34498163

[191] Das D, Singha DL, Paswan RR, Chowdhury N, Sharma M, Reddy PS, Chikkaputtaiah C. Recent advancements in CRISPR/Cas technology for accelerated crop improvement. Planta. 2022;255:109. doi: 10.1007/s00425-022-03894-3. 35460444

[192] Nadakuduti SS, Enciso-Rodríguez F. Advances in genome editing with CRISPR systems and transformation technologies for plant DNA manipulation. Front Plant Sci. 2021;11:637159. doi: 10.3389/fpls.2020.637159. 33519884 PMC7840963

[193] Jaganathan D, Ramasamy K, Sellamuthu G, Jayabalan S, Venkataraman G. CRISPR for crop improvement: an update review. Front Plant Sci. 2018;9:985. doi: 10.3389/fpls.2018.00985. 30065734 PMC6056666

[194] Wang JY, Doudna JA. CRISPR technology: A decade of genome editing is only the beginning. Science (80-.). 2023;379 eadd8643. doi: 10.1126/science.add8643. 36656942

[195] Kubis A, Bar-Even A. Synthetic biology approaches for improving photosynthesis. J Exp Bot. 2019;70:1425–1433. doi: 10.1093/jxb/erz029. 30715460 PMC6432428

[196] Liu J, Friebe VM, Frese RN, Jones MR. Polychromatic solar energy conversion in pigment-protein chimeras that unite the two kingdoms of (bacterio) chlorophyll-based photosynthesis. Nat Commun. 2020;11:1542. doi: 10.1038/s41467-020-15321-w. 32210238 PMC7093453

[197] Baumschlager A. Engineering light-control in biology. Front Bioeng Biotechnol. 2022;10:901300. doi: 10.3389/fbioe.2022.901300. 35573251 PMC9096073

[198] Stano P, Altamura E, Mavelli F, Singh V, Jones DD. Fiat lux! light-driven and light-controlled synthetic biological parts, devices, systems and processes. Front Bioeng Biotechnol. 2023;11:1201962. doi: 10.3389/fbioe.2023.1201962. 37187881 PMC10178064

[199] Brown KA, King PW. Coupling biology to synthetic nanomaterials for semi-artificial photosynthesis. Photosynth Res. 2020;143:193–203. doi: 10.1007/s11120-019-00670-5. 31641988

[200] Schuergers N, Werlang C, Ajo-Franklin CM, Boghossian AA. A synthetic biology approach to engineering living photovoltaics. Energy Environ Sci. 2017;10:1102–1115. doi: 10.1039/C7EE00282C. 28694844 PMC5501249

[201] Sharma A, Kumar V, Shahzad B, Ramakrishnan M, Singh Sidhu GP, Bali AS, Handa N, Kapoor D, Yadav P, Khanna K. Photosynthetic response of plants under different abiotic stresses: a review. J Plant Growth Regul. 2020;39:509–531. doi: 10.1007/s00344-019-10018-x.

[202] Zahra N, Hafeez MB, Kausar A, Zeidi M, Al, Asekova S, Siddique KHM, Farooq M. Plant photosynthetic responses under drought stress: effects and management. J Agron Crop Sci. 2023;209:651–672. doi: 10.1111/jac.12652.

[203] Razi K, Muneer S. Drought stress-induced physiological mechanisms, signaling pathways and molecular response of chloroplasts in common vegetable crops. Crit Rev Biotechnol. 2021;41:669–691. 33525946 10.1080/07388551.2021.1874280

[204] Angon PB, Tahjib-Ul-Arif M, Samin SI, Habiba U, Hossain MA, Brestic M. How do plants respond to combined drought and salinity stress?—A systematic review. Plants. 2022;11:2884. doi: 10.3390/plants11212884. 36365335 PMC9655390

[205] Yusuf M, Khan TA, Saeed T. Epibrassinolide and melatonin co-treatment enhances salt tolerance in tomato plants by coordinating photosynthetic efficiency, proline accumulation, and antioxidant defense. Front Plant Sci. 2025;16:1621310. doi: 10.3389/fpls.2025.1621310. 41041591 PMC12484014

[206] Yusuf M, Saeed T, Almenhali HA, Azzam F, Hamzah AIA, Khan TA. Melatonin improved efficiency of 24-epibrassinolide to counter the collective stress of drought and salt through osmoprotectant and antioxidant system in pea plants. Sci Hortic. 2024;323:112453. doi: 10.1016/j.scienta.2023.112453.

[207] Alyammahi O, Gururani MA. Chlorophyll?a fluorescence analysis reveals differential response of photosynthetic machinery in melatonin?treated oat plants exposed to osmotic stress. Agronomy. 2020;10:1520. doi: 10.3390/agronomy10101520.

[208] Kappachery S, AlHosani M, Khan TA, AlKharoossi SN, AlMansoori N, AlShehhi SAS, AlMansoori H, AlKarbi M, Sasi S, Karumannil S, et al. Modulation of antioxidant defense and PSII components by exogenously applied acetate mitigates salinity stress in avena sativa. Sci Rep. 2024;14:620. doi: 10.1038/s41598-024-51302-5. 38182773 PMC10770181

[209] Patel J, Khandwal D, Choudhary B, Ardeshana D, Jha RK, Tanna B, Yadav S, Mishra A, Varshney RK, Siddique KHM. Differential physio-biochemical and metabolic responses of peanut (Arachis hypogaea L.) under multiple abiotic stress conditions. Int J Mol Sci. 2022;23:660. doi: 10.3390/ijms23020660. 35054846 PMC8776106

[210] Hussain M, Khan TA, Yusuf M, Fariduddin Q. Silicon mediated role of 24-epibrassinolide in wheat under high temperature stress. Environ Sci Pollut Res. 2019;26:17163–17172. doi: DOI 10.1007/s11356-019-04938-0. 31001773

[211] Khan TA, Fariduddin Q, Yusuf M. Low temperature stress: is phytohormones application a remedy? Environ Sci Pollut Res. 2017;24:21574–21590. doi: 10.1007/s11356-017-9948-7. 28831664

[212] Muhammad I, Shalmani A, Ali M, Yang QH, Ahmad H, Li FB. Mechanisms regulating the dynamics of photosynthesis under abiotic stresses. Front Plant Sci. 2021;11:1–25. doi: 10.3389/fpls.2020.615942. PMC7876081 33584756

[213] Khan TA, Yusuf M, Ahmad A, Bashir B, Saeed T, Fariduddin Q, Hayat S, Mock HP, Wu T. Proteomic and physiological assessment of stress sensitive and tolerant variety of tomato treated with brassinosteroids and hydrogen peroxide under low-temperature stress. Food Chem. 2019;289:500–511. doi: 10.1016/j.foodchem.2019.03.029. 30955642

[214] Zait Y, Shtein I, Schwartz A. Long-term acclimation to drought, salinity and temperature in the thermophilic tree ziziphus spina-christi: revealing different tradeoffs between mesophyll and stomatal conductance. Tree Physiol. 2019;39:701–716. 30597082 10.1093/treephys/tpy133

[215] Kurepin LV, Ivanov AG, Zaman M, Pharis RP, Allakhverdiev SI, Hurry V, Hüner NPA. Stress-related hormones and glycinebetaine interplay in protection of photosynthesis under abiotic stress conditions. Photosynth Res. 2015b;126:221–235. doi: 10.1007/s11120-015-0125-x. 25823797

[216] Bianchetti R, Ali A, Gururani M. Abscisic acid and ethylene coordinating fruit ripening under abiotic stress. Plant Sci. 2024;349:112243. doi: 10.1016/j.plantsci.2024.112243. 39233143

[217] Nouri MZ, Moumeni A, Komatsu S. Abiotic stresses: insight into gene regulation and protein expression in photosynthetic pathways of plants. Int J Mol Sci. 2015;16:20392–20416. doi: 10.3390/ijms160920392. 26343644 PMC4613210

[218] Yadav S, Mishra A. Ectopic expression of C4 photosynthetic pathway genes improves carbon assimilation and alleviate stress tolerance for future climate change. Physiol Mol Biol Plants. 2020;26:195–209. doi: 10.1007/s12298-019-00751-8. 32153323 PMC7036372

[219] Lodeyro AF, Krapp AR, Carrillo N. Photosynthesis and chloroplast redox signaling in the age of global warming: stress tolerance, acclimation, and developmental plasticity. J Exp Bot. 2021;72:5919–5937. 34111246 10.1093/jxb/erab270

[220] Sunil B, Saini D, Bapatla RB, Aswani V, Raghavendra AS. Photorespiration is complemented by cyclic electron flow and the alternative oxidase pathway to optimize photosynthesis and protect against abiotic stress. Photosynth Res. 2019;139:67–79. doi: 10.1007/s11120-018-0577-x. 30187303

[221] Rae BD, Long BM, Förster B, Nguyen ND, Velanis CN, Atkinson N, Hee WY, Mukherjee B, Price GD, McCormick AJ. Progress and challenges of engineering a biophysical CO2-concentrating mechanism into higher plants. J Exp Bot. 2017;68:3717–3737. doi: 10.1093/jxb/erx133. 28444330

[222] DePaoli HC, Borland AM, Tuskan GA, Cushman JC, Yang X. Synthetic biology as it relates to CAM photosynthesis: challenges and opportunities. J Exp Bot. 2014;65:3381–3393. doi: 10.1093/jxb/eru038. 24567493

[223] Chan C. Progress in salicylic acid-dependent signaling for growth–defense trade-off. Cells. 2022;11:2985. doi: 10.3390/cells11192985. 36230947 PMC9563428

[224] Anwar A, Kim J-K. Transgenic breeding approaches for improving abiotic stress tolerance: recent progress and future perspectives. Int J Mol Sci. 2020;21:2695. doi: 10.3390/ijms21082695. 32295026 PMC7216248

[225] Laanisto L, Niinemets Ü. Polytolerance to abiotic stresses: how universal is the shade–drought tolerance trade‐off in woody species? Glob Ecol Biogeogr. 2015;24:571–580. doi: 10.1111/geb.12288. 29367836 PMC5777592

[226] Puglielli G, Hutchings MJ, Laanisto L. The triangular space of abiotic stress tolerance in woody species: a unified trade‐off model. New Phytol. 2021;229:1354–1362. doi: 10.1111/nph.16952. 32989754

[227] Cable J, Ronald PC, Voytas D, Zhang F, Levy AA, Takatsuka A, Arimura S, Jacobsen SE, Toki S, Toda E. Plant genome engineering from lab to field—a keystone symposia report. Ann N Y Acad Sci. 2021;1506:35–54. doi: 10.1111/nyas.14675. 34435370

[228] Foyer CH, Ruban AV, Nixon PJ. Photosynthesis solutions to enhance productivity. Philos Trans R Soc B Biol Sci. 2017;372:20160374. doi: 10.1098/rstb.2016.0374. PMC5566875 28808094

[229] Kohli A, Miro B, Balié J, d’A Hughes J. Photosynthesis research: a model to bridge fundamental science, translational products, and socio-economic considerations in agriculture. J Exp Bot. 2020;71:2281–2298. doi: 10.1093/jxb/eraa087. 32076700 PMC7135011

[230] O’Brien CP, Watson MJ, Dowling AW. Challenges and opportunities in converting CO2 to carbohydrates. ACS Energy Lett. 2022;7:3509–3523. doi: 10.1021/acsenergylett.2c01550.

[231] Farooq A, Farooq N, Akbar H, Hassan ZU, Gheewala SH. A critical review of climate change impact at a global scale on cereal crop production. Agronomy. 2023;13:162. doi: 10.3390/agronomy13010162.

[232] Ray DK, West PC, Clark M, Gerber JS, Prishchepov AV, Chatterjee S. Climate change has likely already affected global food production. PLoS One. 2019;14:e0217148. doi: 10.1371/journal.pone.0217148. 31150427 PMC6544233

[233] Heino M, Kinnunen P, Anderson W, Ray DK, Puma MJ, Varis O, Siebert S, Kummu M. Increased probability of hot and dry weather extremes during the growing season threatens global crop yields. Sci Rep. 2023;13:3583. doi: 10.1038/s41598-023-29378-2. 36869041 PMC9984494

[234] Vogel E, Donat MG, Alexander LV, Meinshausen M, Ray DK, Karoly D, Meinshausen N, Frieler K. The effects of climate extremes on global agricultural yields. Environ Res Lett. 2019;14:54010. doi: 10.1088/1748-9326/ab154b.

[235] Wang T, Zhang C, Zhang H, Zhu H. CRISPR/Cas9-mediated gene editing revolutionizes the improvement of horticulture food crops. J Agric Food Chem. 2021;69:13260–13269. doi: 10.1021/acs.jafc.1c00104. 33734711

[236] Aggarwal P, Vyas S, Thornton P, Campbell BM. How much does climate change add to the challenge of feeding the planet this century? Environ Res Lett. 2019;14:43001. doi: 10.1088/1748-9326/aafa3e.

[237] Li K, Pan J, Xiong W, Xie W, Ali T. The impact of 1.5 C and 2.0 C global warming on global maize production and trade. Sci Rep. 2022;12:17268. doi: 10.1038/s41598-022-22228-7. 36241905 PMC9568596

[238] Wheeler T, Braun J, Von. Climate change impacts on global food security. Science (80-). 2013;341:508–513. doi: 10.1126/science.1239402. 23908229

